# Sources of Variation in Food-Related Metabolites during Pregnancy

**DOI:** 10.3390/nu14122503

**Published:** 2022-06-16

**Authors:** Talha Rafiq, Sandi M. Azab, Sonia S. Anand, Lehana Thabane, Meera Shanmuganathan, Katherine M. Morrison, Stephanie A. Atkinson, Jennifer C. Stearns, Koon K. Teo, Philip Britz-McKibbin, Russell J. de Souza

**Affiliations:** 1 Medical Sciences Graduate Program, Faculty of Health Sciences, McMaster University, Hamilton, ON L8S 4L8, Canada; rafiqt2@mcmaster.ca; 2 Population Health Research Institute, Hamilton Health Sciences, McMaster University, Hamilton, ON L8L 2X2, Canada; anands@mcmaster.ca (S.S.A.); koon.teo@phri.ca (K.K.T.); 3 Department of Medicine, McMaster University, Hamilton, ON L8S 4L8, Canada; azabs@mcmaster.ca (S.M.A.); stearns@mcmaster.ca (J.C.S.); 4 Department of Pharmacognosy, Alexandria University, Alexandria 21521, Egypt; 5 Department of Health Research Methods, Evidence & Impact, McMaster University, Hamilton, ON L8S 4L8, Canada; thabanl@mcmaster.ca; 6 Biostatistics Unit, Father Sean O’Sullivan Research Centre, The Research Institute, St Joseph’s Healthcare Hamilton, Hamilton, ON L8N 4A6, Canada; 7 Faculty of Health Sciences, University of Johannesburg, Johannesburg 524, South Africa; 8 Department of Chemistry and Chemical Biology, McMaster University, Hamilton, ON L8S 4M1, Canada; shanmm2@mcmaster.ca (M.S.); britz@mcmaster.ca (P.B.-M.); 9 Department of Pediatrics, McMaster University, Hamilton, ON L8S 4L8, Canada; morriso@mcmaster.ca (K.M.M.); satkins@mcmaster.ca (S.A.A.); 10 Centre for Metabolism, Obesity and Diabetes Research, McMaster University, Hamilton, ON L8S 4K1, Canada; 11 Farncombe Family Digestive Health Research Institute, McMaster University, Hamilton, ON L8S 4K1, Canada

**Keywords:** metabolomics, dietary biomarkers, nutrition, non-dietary factors, confounding, omics, variability, food exposures

## Abstract

The extent to which variation in food-related metabolites are attributable to non-dietary factors remains unclear, which may explain inconsistent food-metabolite associations observed in population studies. This study examined the association between non-dietary factors and the serum concentrations of food-related biomarkers and quantified the amount of variability in metabolite concentrations explained by non-dietary factors. Pregnant women (*n* = 600) from two Canadian birth cohorts completed a validated semi-quantitative food frequency questionnaire, and serum metabolites were measured by multisegment injection-capillary electrophoresis-mass spectrometry. Hierarchical linear modelling and principal component partial R-square (PC-PR2) were used for data analysis. For proline betaine and DHA (mainly exogenous), citrus foods and fish/fish oil intake, respectively, explained the highest proportion of variability relative to non-dietary factors. The unique contribution of dietary factors was similar (15:0, 17:0, hippuric acid, TMAO) or lower (14:0, tryptophan betaine, 3-methylhistidine, carnitine) compared to non-dietary factors (i.e., ethnicity, maternal age, gestational age, pre-pregnancy BMI, physical activity, and smoking) for metabolites that can either be produced endogenously, biotransformed by gut microbiota, and/or derived from multiple food sources. The results emphasize the importance of adjusting for non-dietary factors in future analyses to improve the accuracy and precision of the measures of food intake and their associations with health and disease.

## 1. Introduction

The accurate assessment of dietary intake remains a major challenge in human nutrition research due to the complex nature of food exposure and the reliance on self-reporting, which often leads to biased or unreliable measures of food intake. While most studies use self-reported dietary intake methods, such as food frequency questionnaires (FFQ), 24-h dietary recalls, and food records, they may be subject to recall, misclassification, and measurement biases [1]. To circumvent this problem, metabolomics—the global analysis of low molecular weight metabolites in biological samples—have been increasingly applied in large-scale epidemiological studies for the discovery and validation of food intake biomarkers [2].

Biomarkers can provide a more objective assessment of food exposures than self-reported dietary intake because they account for nutrient bioavailability and metabolism. An ideal biomarker of food intake is one that can be readily measured in human biofluid (blood or urine) at the population level, is highly specific for one food item or food group, shows a dose- and time-dependent response, and is not extensively transformed by the microbiota and host tissue upon consumption. However, complex interpretative challenges exist since nutrients are derived from various food sources and can display intercorrelation between other metabolic processes [3]. Furthermore, the human metabolome exhibits variability due to intrinsic physiologic characteristics, such as age, sex, hormonal levels, and the gut microbiome, as well as due to extrinsic factors, such as habitual diet and lifestyle. Further, many putative biomarkers of food intake do not exclusively originate from a single food or nutrient. For example, trimethylamine *N*-oxide (TMAO) is formed from a TMA-containing nutrient, such as choline, which is abundant in fish, beef, and eggs, but can also be produced from carnitine in red meat [2,4]. Moreover, many of the gut-microbiome-dependent metabolites and other food-specific metabolites are metabolized in the liver at different rates, depending on hepatic enzyme activity [5], which may contribute to the greater variability observed in the range of metabolite measured in the biological samples [6]. Consequently, it is important to identify potential non-dietary sources of food-related biomarkers and examine the extent to which these factors explain differences in metabolite concentration.

In most cases, food intake explains a relatively small proportion (R^2^ < 10%) of the total variation in a given metabolite concentration, and other determinants are typically unknown, unmeasured, or, if measured, the extent of the measurement error is not clear [7]. Biomarkers derived from food intake and gut microbiota are influenced by non-dietary factors [8,9]; however, the extent to which these factors compromise the validity of the metabolite as a food intake biomarker may depend on the specificity of the biomarker (well-established, uncertain, or weak biomarker of the particular food), whether the biomarker is endogenously produced, biotransformed by gut microbiota, and/or derived from more than one food source. Understanding the sources of variation in biomarkers of food intake that are not attributed to changes in food intake are critical to advancing the application/field of food intake biomarkers. If the sources of the variation are not clearly understood, then using these biomarkers as markers of food/nutrient intake may simply exchange one source of measurement error (self-misreport) for others (changes in the biomarker intake unrelated to changes in food intake).

Carefully designed studies examining the association between non-dietary factors and biomarker concentrations are sparse and especially lacking in women during pregnancy. Observational studies, specifically birth cohort studies, are useful designs to learn about pregnancy exposures and birth outcomes [10]. Women experience a series of metabolic modifications during pregnancy, likely affected by pre-pregnancy and intrapartum factors, which in turn may affect maternal health and disease at the critical stages of fetal development [11,12]. Moreover, metabolite concentrations during gestation and pre-pregnancy, and pregnancy-related factors, such as GDM, also differ between ethnic groups (e.g., White Europeans and South Asians) [9]. The purpose of this study was to examine the associations of non-dietary factors, including demographics, lifestyle, and pregnancy-related factors with serum metabolite concentrations using a panel of commonly identified biomarkers derived from food intake and/or gut microbiota, including proline betaine, five fatty acids (even-chain saturated fatty acids (SFA) myristic acid (14:0); odd-chain SFA pentadecanoic acid (15:0) and heptadecanoic acid (17:0); omega-3 polyunsaturated fatty acids (ω-3 PUFA), docosahexaenoic acid (DHA) and eicosapentaenoic acid (EPA); hippuric acid; TMAO; 3-methylhistidine; carnitine; and tryptophan betaine, in pregnant women of two ethnically diverse groups, and to determine the extent to which non-dietary factors explain the variability in the concentrations of the putative biomarkers of food intake.

## 2. Materials and Methods

### 2.1. Data Source and Participants

This study used data from two longitudinal Canadian birth cohorts of pregnant women: the Family Atherosclerosis Monitoring In earLY life (FAMILY) study and SouTh Asian biRth cohorT (START). The FAMILY study included White European women and the START cohort included women of South Asian ethnic background. Design and methodology of these two studies have been described in detail elsewhere [13,14]. Briefly, the FAMILY study was designed to understand the environmental, genetic, and biochemical factors important in the development of obesity and cardiovascular disease risk factors in childhood. A total of 857 families (901 newborns) were recruited between 2002 and 2009 in the Hamilton area, Ontario, Canada. Women were recruited between 24 and 36 weeks of gestation. The START study enrolled 1012 South Asian (people who originate from the Indian sub-continent: India, Pakistan, Sri Lanka, or Bangladesh) mother–child pairs between 2011 and 2015 from the Peel Region of Ontario to investigate the influence of diverse environmental exposures and genetics on early life adiposity, growth trajectory, and cardiometabolic risk. The ancestral origin of both the woman, her partner, and both offspring’s grandparents were required to be classified as South Asian.

All enrolled participants provided full informed consent, and both studies obtained ethics approval from the McMaster Hamilton Integrated Research Ethics Board [START (HiREB #10–640) and FAMILY (HiREB #02–060)].

Clinical and demographic data was harmonized across the two cohorts. When questions were not identical between studies (e.g., physical activity level during pregnancy), comparable categories were constructed with the available data to satisfy the same definition. Within each cohort, 300 pregnant women were randomly selected for serum metabolomics analysis as previously described [15]. This selection was based on the contrasting diet quality score (DQS), where 100 mothers were randomly selected from the 3 DQS groups (>90th percentile [“high” diet quality], <10th percentile [“low” diet quality], and between 10th and 90th percentile [“intermediate” diet quality]). A total of 600 pregnant mothers were included in the current analysis (Supplementary Figure S1).

### 2.2. Maternal Serum Metabolome Analyses

A validated multiplexed separation platform based on multisegment injection–capillary electrophoresis–mass spectrometry (MSI-CE-MS) was used for targeted and nontargeted profiling of polar/ionic metabolites measured consistently in serum filtrate samples with stringent quality control (QC). A standardized method protocol was used for the identification and quantification of the maternal serum metabolome, as described in more detail elsewhere [15]. Briefly, a total of 66 and 67 polar ionic metabolites from serum filtrate samples satisfied selection criteria for their analysis in the FAMILY and START cohorts, respectively, and 53 of these were measured consistently across both cohorts. Serum metabolites were reported only if they satisfied two additional criteria: (1) metabolites that were detected in the majority of the individual samples (≥75%) in a cohort (i.e., frequency filter) and (2) acceptable technical precision based on the repeated analysis of QC samples (i.e., QC filter) to reduce false discoveries and data overfitting. Metabolites with nondetectable or missing values were replaced with half of the lowest detected value for the compound in each cohort. Moreover, a QC-based batch correction algorithm was applied for the robust correction of long-term monitoring of signal drift in MSI-CE-MS [16]. Among metabolites measured consistently in the two cohorts, six metabolites, including proline betaine, 3-methylhistidine, hippuric acid, TMAO, carnitine, and tryptophan betaine, were selected for our current analysis, as they were previously determined to be associated with the self-report of dietary intake [2,17]. Further, they offer a combination of evidence (good, fair, or poor) for candidate biomarkers of food intake that are produced exogenously, endogenously, biotransformed by gut microbiota, and/or derived from more than one food source [2,17,18]. The reference interval for these serum metabolites in different birth cohorts from across Canada, their technical/biological variance, and interclass correlation coefficients have been reported previously [15].

Non-esterified fatty acid (NEFAs) from serum ether extracts were analyzed in the FAMILY cohort using a validated protocol based on MSI-NACE-MS, which offers a multiplexed separation platform for rapid NEFA analysis on an Agilent 6230 TOF mass spectrometer with a coaxial sheath liquid ESI ionization source equipped with an Agilent G7100A capillary electrophoresis (CE) (Agilent Technologies Inc., Santa Clara, CA, USA) [19]. Serum extracts were injected with alternating background electrolyte spacer plugs for a total of seven discrete samples analyzed within 30 min for a single run. Repeat QC samples introduced in a randomized position for each MSI-NACE-MS run were analyzed for NEFA confirming technical precision (mean CV = 15%, *n* = 46). Serum non-esterified 14:0, 15:0, 17:0, DHA, and EPA were reliably quantified and reported as relative proportions as a percentage by moles of a total quantified of 19 NEFAs (mol%) [20].

### 2.3. Assessment of Dietary Intake

Maternal dietary intake during pregnancy was collected at 24–28 weeks gestation. Semi-quantitative validated food-frequency questionnaires (157 items in the FAMILY and 163-items in the START) developed and validated as part of the Study of Health and Risk in Ethnic Groups (SHARE) Study were used [21,22,23]. Participants were asked to report on the frequency (daily, weekly, monthly, yearly, or never) and amount in serving size of each food or food group on average in the past 12 months. For our study, food items were either used as separate items (chicken, canned fish, fried fish) or classified into main food groups: citrus food (citrus fruit and citrus juice), red meat, eggs (boiled and fried eggs), seafood, nuts and legumes, and fruits and vegetables. Nutrient intake was calculated using the ESHA Food Processor Nutrient Analysis Software (ESHA Research, version 6.11, 1996, Salem, OR, USA), derived from the 1991 Canadian Nutrient File and the US Department of Agriculture nutrient food composition databases. Fiber intake and total energy intake was also estimated using the FFQ [17,21]. Data were logarithm-transformed to correct for skewness prior to including them in the regression analysis, and nutrient intakes were adjusted for energy intake using the residual approach [24].

### 2.4. Non-Dietary Factors

Non-dietary factors included ethnicity (White European or South Asian), maternal age (years), gestational age (i.e., weeks of pregnancy), parity, pre-pregnancy body mass index (BMI, kg/m^2^), smoking history (current or former smoker and never smoker), physical activity (mainly sedentary, mild activity, moderate activity, and strenuous activity), social disadvantage index (SDI), and gestational diabetes (GDM). For SDI, derived using a previously validated index based on employment status, income, and marital status, higher values indicate greater socioeconomic disadvantage [25]. A case of GDM was defined based on the Born in Bradford (BiB) oral glucose tolerance test criteria, self-reported GDM, and insulin use in pregnancy in START cohort, whereas the International Association of the Diabetes and Pregnancy Study Groups (IADPSG) criteria (75 g OGTT with fasting glucose ≥ 5.1 mmol/L, 1 h ≥ 10.0 mmol/L, 2 h ≥ 8.5 mmol/L) was used in the FAMILY cohort. We selected these factors based on the known and plausible associations with the selected metabolites and/or the fact that they are commonly adjusted in population-based nutritional metabolomics studies.

### 2.5. Statistical Analysis

Descriptive statistics for categorical variables were summarized using frequency and percentages, and continuous data were summarized using mean and standard deviation (SD) or median and interquartile range (IQR). Random-effects hierarchical linear models (HLM) were fit, whereby each of the natural logarithm-transformed food-metabolite concentration was regressed on dietary and non-dietary factors after adjusting for other covariates, including total energy intake (kcal), total fiber intake (g/day), and period of time between the day FFQ information was collected and blood was drawn (FFQ before blood, FFQ after blood, and both taken on the same day) [26].

The data had a nested (clustered) structure, where individuals within the same cohort represented a cluster because they were more similar to one another with regards to dietary and non-dietary factors. Therefore, we used hierarchical linear modeling (HLM) to accommodate the dependent nature of observations in clustered data. HLM allows nesting effects to be incorporated into the model, producing more accurate estimates and corrects for the error structure violations (non-independent errors) to provide robust conclusions [27,28]. First, unconditional (intercept-only) HLM models were tested to determine whether serum metabolite concentrations were nested within cohort using an intraclass correlation (ICC) calculated based on the covariance parameter estimates. An ICC refers to the amount of variation attributed to level-two (study-level) factor. An ICC can be determined from an intercept-only model and any relationship with an ICC of 2% or greater suggests the presence of level 2 effects [26]. The results showed an ICC of 3.9% for proline betaine, 25.6% for 3-methylhistidine, 1.5% for carnitine, 0% for hippuric acid, 46.0% for tryptophan betaine, and 7.0% for TMAO. A sensitivity analysis using an ordinary least squares (OLS) multivariable linear regression was conducted for carnitine and hippuric acid (Supplementary Table S1). Next, the association between dietary factors as level 1 predictors (fixed), previously shown to be associated with a specific metabolite (e.g., citrus fruit and proline betaine), was examined. Finally, in addition to the dietary factors, all non-dietary factors were also added as level 1 predictors. These HLM procedures produced the following three models:

**Intercept-only Model (Unconditional Model)**

Metabolite*ij* = *β*0*j* + *eij*
*β*0*j* = *γ*00 + *u*0*j*

**Random Intercept (*u*0** ***j*) with Fixed Level 1 Factors (Dietary factors, *γ*10)**

Metabolite*ij* = *β*0*j* + *β*1*j***Dietaryfactor** + *eij*
*β*0*j* = *γ*00 + *u*0*j*
*β*1*j* = *γ*10

**Random Intercept (*u*0** ***j*) with Fixed Level 1 Factors (Dietary (*γ*10) and Non-dietary factors (*γ*20…))**

Metabolite*ij* = *β*0*j* + *β*1*j***Dietaryfactor** + *β*2*j***Age …** + *eij*
*β*0*j* = *γ*00 + *u*0*j*
*β*1*j* = *γ*10
*β*2*j* = *γ*20

The goodness-of-fit statistics, including the Akaike Information Criterion [AIC], Bayesian Information Criterion [BIC] and the change in deviance statistics, were used to evaluate model fit in terms of the clustering variable. Smaller values of these statistics indicate a better model fit [29]. The AIC and BIC consider error and model parsimony simultaneously. An OLS multivariable linear regression was conducted for NEFAs as these data were only available in the FAMILY cohort. Regression estimates of (b) 95% confidence intervals (95% CI) and *p*-values were reported, and statistical analysis was conducted using SAS software version 9.4.

Finally, principal component partial R-square (PC-PR2) analysis was used to quantify the sources of systematic variability in serum metabolite concentrations [30]. The PC-PR2 method combines features of principal component analysis (PCA) and the partial R-square statistic in multivariable linear regression and allows for some degree of inter-correlation between explanatory variables. The mathematical details of the PC-PR2 method are described elsewhere [30]. A data reduction component was not necessary because the analytic strategy was applied to a single metabolite. The partial R^2^ statistic was calculated for each explanatory variable, which quantifies the amount of variability in metabolite explained by that variable, conditional on all other covariates included in the model. The PC-PR2 method was conducted using the R software, version 1.2.5.

## 3. Results

### 3.1. Association of Dietary and Non-Dietary Factors with Food-Related Metabolites

The descriptive characteristics of the participants overall and by ethnicity are shown in Table 1. Model fit statistics from the HLM examining the dietary and non-dietary factors associated with food-intake biomarkers are presented in Supplementary Table S2, and the regression estimates and 95% CI are presented in Table 2. Three regression models, including an unconditional model (Model 1), the random intercept model with level 1 dietary factors (Model 2), and random intercept model with level 1 dietary and non-dietary factors (Model 3), were examined (Supplementary Table S2). For each metabolite outcome, the log likelihood, AIC, and BIC statistics decreased considerably after adding the non-dietary covariates, indicating better model fit. Thus, the regression estimates presented in Table 2 are based on Model 3. As expected, most of the dietary food sources were significantly associated with their respective metabolite concentrations, except for carnitine (*p* > 0.05) (Table 2). For exogenous metabolites specific to a single food source, higher citrus food intake was positively associated with proline betaine concentration (b: 0.27; 95% CI: 0.20, 0.34), and a higher intake of nuts and legumes was positively associated with tryptophan betaine concentration (b: 0.02; 95% CI: 0.00, 0.03). For metabolites with both endogenous metabolic and exogenous sources and obtained from multiple food sources, such as hippuric acid, higher intake of fruits and vegetables were associated with higher hippuric acid concentration (b: 0.22; 95% CI: 0.08, 0.36), but no such association was found with tea and coffee intake. Higher intake of chicken (b: 0.02; 95% CI: 0.00, 0.04) and red meat (b: 0.03; 95% CI: 0.01, 0.06) were positively associated with 3-methyl-histidine concentration, while seafood intake was positively associated with TMAO concentration (b: 0.08; 95% CI: 0.04, 0.12) (Table 2).

For non-dietary factors, maternal age, gestational age, and smoking history were associated with the serum concentration of some metabolites after adjusting for diet-related factors (Table 2). Higher maternal age was associated with a higher concentration of proline betaine (b: 0.04; 95% CI: 0.01, 0.07) and TMAO (b: 0.02; 95% CI: 0.00, 0.04), and higher gestational age of pregnancy was associated with a higher concentration of 3-methyl-histidine (b: 0.01; 95% CI: 0.00, 0.02) and lower concentration of carnitine (b: −0.01; 95% CI: −0.02, −0.01). Participants who indicated having ever smoked cigarettes had a lower concentration of proline betaine (b: −0.60; 95% CI: −0.95, −0.25) and a higher concentration of carnitine (b: 0.06; 95% CI: 0.02, 0.10) compared to those who never smoked cigarettes (Table 2). Parity, GDM, pre-pregnancy BMI, physical activity, SDI, and the timing of the administration of the FFQ (before or after the blood draw relative to at the same time as the blood draw) were found to not be associated with any of the six metabolite concentration outcomes. The results for the HLM models examining the association of dietary and non-dietary factors with food-related metabolites stratified by ethnicity (White European and South Asians) are presented in Supplementary Tables S3 and S4, respectively. The results between the two cohorts were generally similar to those reported for the overall sample.

The results from the OLS regression models examining the association of dietary and non-dietary factors with NEFAs are presented in Table 3. Higher intake of full-fat dairy was positively associated with odd-chain SFAs 15:0 (b: 0.06; 95% CI: 0.03, 0.10) and 17:0 (b: 0.04; 95% CI: 0.01, 0.07), and higher fish/fish oil daily servings were positively associated with DHA (b: 0.11; 95% CI: 0.07, 0.14) and EPA + DHA (b: 0.08; 95% CI: 0.04, 0.12). For non-dietary factors, higher gestational age of pregnancy was associated with lower odd-chain SFAs 15:0 (b: −0.02; 95% CI: −0.03, −0.01) and 17:0 (b: −0.01; 95% CI: −0.02, −0.01), higher pre-pregnancy BMI was associated with both lower percentage concentrations (mol%) of even-chain and odd-chain SFAs 14:0, 15:0, and 17:0 (b: −0.01; 95% CI: −0.02, −0.00) and lower DHA (b: −0.01; 95% CI: −0.02, −0.00), and higher physical activity was associated with lower 17:0 (b: −0.10; 95% CI: −0.17, −0.02). The results examining the association of dietary fish intake and ω−3 PUFA are presented in Supplementary Table S5.

### 3.2. Results from PC-PR2 Analysis

**Figure 1:**
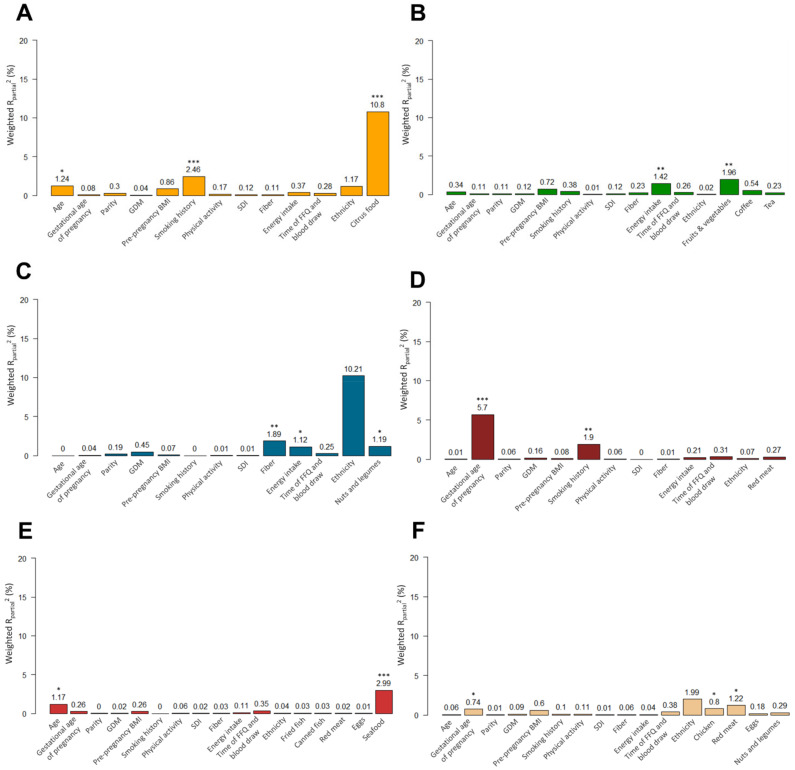
Weighted R_partial_^2^ for each factor showing the percentage of explained variability in: (**A**) Proline betaine, (**B**) Hippuric acid, (**C**) Tryptophan betaine, (**D**) Carnitine, (**E**) trimethylamine *N*-oxide (TMAO), and (**F**) 3-methylhistidine. Statistical significance was based on hierarchical linear models. * *p* ≤ 0.05, ** *p* ≤ 0.01, *** *p* ≤ 0.001. Intraclass correlation suggested a cluster effect by ethnicity (level 2 factor) for proline betaine (ICC = 3.9%), tryptophan betaine (ICC = 46.0%), TMAO (ICC = 7.0%), and 3-methylhistidine (ICC = 25.6%) and did not suggest a cluster effect by ethnicity for hippuric acid (ICC = 0.0%) and carnitine (ICC = 1.5%).

**Figure 2:**
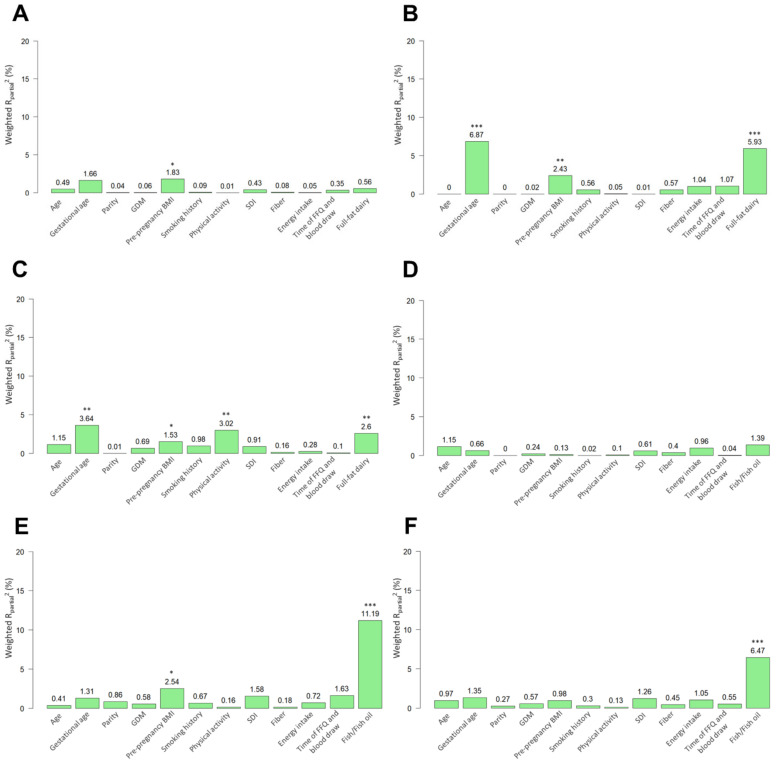
Weighted R_partial_^2^ for each factor showing the percentage of explained variability in: (**A**) Myristic acid (14:0), (**B**) Pentadecanoic acid (15:0), (**C**) Heptadecanoic acid (17:0), (**D**) Eicosapentaenoic acid (EPA, 20:5n-3), (**E**) Docosahexaenoic acid (DHA; 22:6n-3), and (**F**) EPA + DHA in FAMILY cohort. Statistical significance was based on ordinary least squares regression. * *p* ≤ 0.05, ** *p* ≤ 0.01, *** *p* ≤ 0.001.

PC-PR2 analysis was utilized to quantify the sources of systematic variability in serum metabolite concentrations, and the results for the overall sample are displayed in Figure 1 and Figure 2, and results stratified by cohort are displayed in Supplementary Figures S2–S7. For largely exogenous metabolites, such as proline betaine, hippuric acid, and tryptophan betaine, dietary food intake explained a greater proportion of variability in the metabolite than non-dietary factors. Citrus fruit intake explained the largest proportion of variation in proline betaine concentration with a R^2^_partial_ value of 10.8%, followed by smoking history (2.5%), maternal age (1.2%), and ethnicity/cohort (1.2%) (Figure 1A). Similarly, for hippuric acid, fruits and vegetables intake displayed the largest R^2^_partial_ value of 2.0%, followed closely by energy intake (1.4%) (Figure 1B). For tryptophan betaine, intake of nuts and legumes, fiber intake, and overall energy intake explained between 1.2% and 1.9% of the variability. Meanwhile, ethnicity has quite a substantial impact on tryptophan betaine levels as the R^2^_partial_ value of cohort was 10.2% (Figure 1C). When the model was stratified by cohort, nuts and legumes explained the most variability (3.6%) in the FAMILY cohort (primarily White European women), while fiber intake (4.2%), energy intake (2.2%), and GDM (1.5%) explained most of the variability in tryptophan betaine in the START cohort (exclusively South Asian women) (Supplementary Figure S4).

For endogenous (less food-specific) metabolites, the dietary factors explained the most variability for two of the metabolites (3-methyl-histidine and TMAO), while non-dietary factors, such as gestational age (R^2^_partial_ value: 5.7%) and smoking history (R^2^_partial_ value: 1.9%), appeared to play a more prominent role in explaining the variability in carnitine (Figure 1D). This latter finding is also consistent with the results obtained from HLM showing no dietary factor was associated with carnitine concentration. Seafood intake explained the greatest proportion of variability in TMAO, with a R^2^_partial_ value of around 3.0%, followed by maternal age (R^2^_partial_ value: 1.2%) (Figure 1E). For 3-methyl-histidine, red meat intake had the highest R^2^_partial_ value of 1.2% (Figure 1F). There was evidence of differences by ethnicity/cohort, where red meat explained 5.8% of the variability in 3-methyl-histidine in the START cohort but a negligible amount in the FAMILY cohort. Each of the remaining explanatory variables explained a negligible amount of total variation in the metabolite concentrations. Although there were some differences in findings between the two cohorts, overall, the results obtained from PC-PR2 are congruent with those obtained from the HLM analysis.

For NEFAs, pre-pregnancy BMI (R^2^_partial_ value: 1.8%) explained the most variability in even-chain SFA 14:0 (Figure 2A). Gestational age explained the most variability in odd-chain SFAs 15:0 (R^2^_partial_ value: 6.9%) and 17:0 (R^2^_partial_ value: 3.6%), followed by full-fat dairy intake (R^2^_partial_ value: 5.9%) and pre-pregnancy BMI (R^2^_partial_ value: 2.4%) for 15:0, and physical activity (R^2^_partial_ value: 3.0%) and full-fat dairy intake (R^2^_partial_ value: 2.6%) for 17:0 (Figure 2B,C). Fish/fish oil intake explained the greatest proportion of variability in DHA (R^2^_partial_ value: 11.2%), followed by pre-pregnancy BMI (R^2^_partial_ value: 2.5%) (Figure 2E).

## 4. Discussion

Using data from two birth cohorts representing two ethnically diverse groups, the results showed that for exogenous biomarkers such as proline betaine and (largely) DHA, dietary factors explained higher proportion of variability whereas the contribution of nondietary factors was relatively little. On the contrary, for metabolites that can either be produced endogenously, biotransformed by gut microbiota, and/or derived from more than one food source, the unique contribution of dietary factors was similar (15:0, 17:0, hippuric acid, and TMAO) or lower (14:0, tryptophan betaine, 3-methylhistidine, and carnitine) compared to non-dietary factors (ethnicity, maternal age, gestational age, pre-pregnancy BMI, physical activity, and smoking history). Further, there was an ethnicity effect for all metabolites, except carnitine and hippuric acid.

For the non-dietary factors, higher maternal age was positively associated and ever having smoked was inversely associated with proline betaine concentrations after adjusting for citrus foods. Evidence indicates that older women are more likely to make healthier choices including increasing their consumption of fruits and vegetables from pre-pregnancy to pregnancy compared to younger women [31,32]. Many studies have also shown that smokers have lower concentrations of antioxidants and elevated concentration of 8-isoprostane [33,34], which may be due to low consumption of antioxidants [35], reduced vitamin C absorption, or decreased turnover of vitamin C by free radicals produced from smoking [36]. Proline betaine (stachydrine), a marker of citrus foods, which are rich in vitamin C (potent water-soluble antioxidant), has been shown to inhibit cell proliferation and production of reactive oxygen species in in vitro and in vivo studies [37,38]. As expected, higher citrus food intake was associated with proline betaine concentration and explained the largest proportion of variation in proline betaine concentration relative to non-dietary factors. In kinetics studies, proline betaine is excreted rapidly and nearly completely in urine within 24 h [39], and therefore it is considered to be minimally metabolized in humans. Furthermore, proline betaine was previously validated in a large-scale observational study, where it was highly sensitive (86.3%) and specific (90.6%) for citrus fruit consumption [39], and thus considered a robust biomarker for citrus food intake.

Even-chain SFA (14:0) can be derived from both exogenous sources (via dietary intake) and endogenous synthesis (via de novo lipogenesis) [40,41], whereas odd-chain SFAs (15:0 and 17:0) mainly reflect dietary intake of full-fat dairy [42], though the possible contribution of endogenous sources cannot be ruled out [43,44]. As expected, both 15:0 and 17:0 were associated with full-fat dairy intake and 14:0 was not. Full-fat dairy intake did not, however, explain the largest variance in 15:0 or 17:0 levels. Rather, non-dietary factors, including higher gestational age and pre-pregnancy BMI, were associated with lower odd chain SFA (15:0 and 17:0) and low physical activity level was associated with lower 17:0. In a previous longitudinal analysis, odd-chain SFA (sum of 15:0 and 17:0) progressively declined during pregnancy [45]. Although the exact mechanism for the gestational alterations in these SFAs remain unclear, it is possible that pregnancy associated physiologic changes and increase in adipose deposition throughout pregnancy may be important factors contributing to the observed differences [46]. In several population-based studies, higher circulating odd-chain SFAs (15:0 and 17:0) were inversely associated with obesity and cardiometabolic diseases [47,48]. ω-3 PUFAs (DHA more than EPA) have been considered robust biomarkers of habitual fish/fish oil intake [2]. This association was demonstrated for DHA in the current study where fish/fish oil intake explained the largest proportion of variation in DHA relative to non-dietary factors. Fish/fish oil daily servings explains about twice the amount of variation in ω-3 PUFAs compared with dietary fish intake, indicating that it is important to account for EPA and DHA sources from both diet and supplements.

For other metabolites, non-dietary factors were associated with metabolite concentrations, however, their overall contribution was minimal, except for carnitine which was mostly explained by gestational age. Carnitine mainly reflects the consumption of amino acids and fatty acid-containing foods and, as a result, is considered a generic marker for foods of animal origin but may also be synthesized from the essential amino acids lysine and methionine [4,49]. A decline in carnitine across trimesters during pregnancy was previously reported [9,50]. A significant rise in acylcarnitine in pregnant women as pregnancy progresses may reflect enhanced fatty acid oxidation in later periods of gestation [50]. This distribution may suggest a greater uptake of carnitine in the fatty acid β-oxidation process, leading to a lower free carnitine substrate and resulting in a lower total body carnitine pool in pregnant women [51,52].

For all metabolites, except for proline betaine and two NEFAs (15:0 and DHA), the unique contribution of food sources was similar to or lower than non-dietary factors. This may reflect endogenous production, microbial synthesis, or the multiple food sources of some of these metabolites. Interindividual variability in hippuric acid [53,54], TMAO [55,56], and tryptophan betaine [57,58] may partly be due to differences in intestinal microbiota. However, the potential variation in these metabolites attributable to the gut microbiome could not be accounted for in our study. Further, variation in an endogenous metabolite concentration such as carnitine may reflect the general intake of foods of animal origin and/or physiological changes that take place during pregnancy, and is influenced by factors such as age and health status, and thus may not be a suitable biomarker of red meat at the population level [4,49].

Metabolite concentration may also vary widely across cultures and ethnic groups as the type of food, method of consumption, and food preparation techniques may vary [59]. In our multi-level analysis, there was an ethnicity effect for all metabolites, except carnitine and hippuric acid. Proline betaine concentration was shown to vary to some extent by cohort, likely attributable to differences in citrus food intake in the two cohorts (Table 1). Additionally, some of this variability may be attributed to differences in lifestyle factors between members of the two cohorts, such as smoking status. Regardless, citrus fruit consumption still explained the largest amount of variance in proline betaine in both cohorts, suggesting that non-dietary factors do not contribute substantially to proline betaine variation (Supplementary Figure S2). However, mixed results were shown by cohort for metabolites that are synthesized or modified by gut bacteria. Tryptophan betaine concentration was shown to vary considerably between the two cohorts, with higher tryptophan betaine associated with higher nuts and legumes intake in the FAMILY cohort, and with higher fiber intake and lower kilocalories in the START cohort. A possible explanation for this discrepancy may be that nuts and legumes is a heterogeneous food group so the type of nuts and preparation/cooking methods for legumes may play an important role [60]. Further, it is also likely that the association of nuts or legumes intake with tryptophan betaine may be confounded by fiber intake in the START cohort, as fiber intake is higher in this cohort, and tryptophan betaine has been identified in fiber-rich plant-based foods and linked to gut microbiota in fiber-enriched diets [57].

Hippuric acid was one of the metabolites that did not vary by ethnic cohort but was only associated with greater fruit and vegetable intake in the FAMILY cohort despite greater intake in the START cohort. An explanation for this may be related to the metabolism of different dietary polyphenols [61]. Evidence suggests that differences in excretion of hippuric acid may reflect altered gut microbial metabolism [62]. Generally, the amount of variability in food consumption may also affect the robustness of the association. For example, the IQR for certain foods, such as chicken and red meat, were higher in FAMILY compared to START, whereas variability for other foods, such as fruits and vegetables, tea, eggs, and nuts and legumes, were higher in START compared to FAMILY. This may explain inconsistencies in the results for at least some serum metabolites, such as the association between red meat and TMAO in the START cohort.

In other comparisons, TMAO varied slightly by cohort, but this may be explained by a relatively lower consumption of meats, including red meat, canned and fried fish, and seafood, in the START cohort compared to the FAMILY cohort. Despite this, higher seafood intake was positively associated with TMAO concentration in both cohorts. Differences in TMAO production and excretion may partly be related to metabolic precursors, such as choline, betaine, and carnitine. TMAO concentration increases postprandially (within 15 min) after the consumption of fish [63], but it takes more time after consumption of meat [64], suggesting that free TMAO in seafood may be readily absorbed after fish consumption without much involvement of gut microbiota. Finally, although the association of 3-methylhistidine with chicken and red meat was significant in the overall sample, these associations were attenuated when analysis was stratified by cohort. This is likely because the intra-cohort variability was small or because intakes of these foods were highly correlated (as was the case in the START cohort).

Finally, biomarkers with ‘good’ evidence are considered as direct surrogates for food intake [65]. However, there are several factors, in addition to food exposure, that can influence variation in food-related metabolites concentration and thus require appropriate consideration during the statistical analyses of the data [66]. In line with previous research [7], in most cases, our study found that dietary factors explained less than 10% of the total variation in metabolite concentration. While some aspects of the source of the errors are explained by measurement error (self-report), others can be related to non-dietary factors. Therefore, future studies should account for non-dietary factors and differences by ethnicity to control for some of the inter-individual variation in food-related metabolites.

Our study has several strengths, including a large sample size that allowed for stratification by ethnicity, use of fasting serum samples, and comparing a diverse set of metabolites reflecting commonly consumed foods which have been previously reported in free-living population studies [2]. We adopted a novel methodological approach to address an unanswered question regarding the non-dietary sources of metabolite variation in the field of nutritional metabolomics and biomarkers of food intake. Our study also has some limitations. We included only pregnant women from white European and South Asian backgrounds, and thus the generalizability of our findings is limited to these populations. Dietary assessment was based on a self-reported FFQ and may be prone to some measurement error; however, FFQs are commonly used in nutritional epidemiology. The period of dietary assessment of 12 months may not be indicative of recent intake of foods or intake of foods only during pregnancy, but since our aim was to identify sources of variability in metabolites of foods that reflect habitual dietary intake, a 12-month intake was more appropriate. Samples were collected at one point in pregnancy and data on changes in dietary intake during pregnancy were not collected and, therefore, not available for the analysis.

## 5. Conclusions

Overall, the results emphasize that serum metabolites that reflect specific foods are also influenced by non-dietary factors (ethnicity, maternal age, gestational age, pre-pregnancy BMI, physical activity, and smoking history) but to differing degrees. The results of this study provide insight into the external factors that impact serum metabolite concentrations and provide guidance on appropriate modeling when metabolomics is used in nutritional epidemiological studies to identify diet-disease associations. Identifying robust and generalized food related biomarkers in diverse populations remains a challenge, but appropriate adjustment for non-dietary factors is necessary for an unbiased assessment of metabolite concentration. Future work will explore the role of maternal nutrition and food exposures on health outcomes later in life, such as childhood obesity and metabolic syndrome.

## Figures and Tables

**Table 1:** Descriptive statistics of participants overall and by ethnicity.

Factor	Overall *n* = 600	White European *n* = 300	South Asian *n* = 300	*p*-Value
**Age (years), mean (SD)**	31.20 (4.50)	32.35 (4.89)	30.01 (3.73)	<0.0001
**Gestational age (weeks), mean (SD)**	28.06 (3.27)	29.50 (3.76)	26.61 (1.75)	<0.0001
**Pre-pregnancy BMI (kg/m^2^), mean (SD)**	25.35 (5.63)	26.77 (6.39)	23.94 (4.33)	<0.0001
**Parity, *n* (%)**				
**0**	240 (42.33)	145 (48.33)	95 (35.58)	0.0528
**1**	229 (40.39)	110 (36.67)	119 (44.57)	
**2**	76 (13.40)	34 (11.33)	42 (15.73)	
**≥3**	22 (3.88)	11 (3.67)	11 (4.12)	
**Gestational diabetes (GDM), *n* (%) *^a^***	169 (28.94)	50 (17.54)	119 (39.80)	<0.0001
**Smoking history (ever smoked), *n* (%)**	104 (17.48)	104 (35.25)	0 (0.00)	<0.0001
**Physical activity (moderate/vigorous), *n* (%)**	144 (24.04)	84 (28.00)	60 (20.07)	0.0231
**Social disadvantage index, mean (SD) *^b^***	1.31 (1.37)	0.85 (1.22)	1.84 (1.35)	<0.0001
**Fiber intake (g/day), mean (SD)**	22.52 (10.24)	20.66 (9.23)	24.38 (10.85)	<0.0001
**Energy Intake (kcal), mean (SD)**	2165.39 (772.06)	2327.86 (766.33)	2002.92 (744.26)	<0.0001
**Time of FFQ and blood draw, *n* (%)**				
**FFQ and blood draw on same day**	354 (60.31)	88 (29.33)	266 (92.68)	<0.0001
**FFQ before blood draw *^c^***	221 (37.65)	206 (68.67)	15 (5.23)	
**FFQ after blood draw *^c^***	12 (2.04)	6 (2.00)	6 (2.09)	
**Food items (servings/day), median (IQR)**				
**Citrus food**	0.57 (0.95)	0.64 (0.99)	0.43 (0.89)	<0.0001
**Fruits and vegetables**	6.28 (5.74)	5.12 (4.26)	7.85 (6.06)	<0.0001
**Tea**	0.43 (0.98)	0.14 (0.57)	1.0 (1.36)	<0.0001
**Coffee**	0 (0.14)	0.02 (0.64)	0 (0.00)	<0.0001
**Canned fish**	0 (0.03)	0.03 (0.07)	0 (0.00)	<0.0001
**Fried fish**	0 (0.03)	0.01 (0.03)	0 (0.02)	<0.0001
**Seafood**	0 (0.01)	0.01 (0.02)	0 (0.00)	<0.0001
**Chicken**	0.10 (0.29)	0.14 (0.21)	0 (0.14)	<0.0001
**Eggs**	0.21 (0.40)	0.20 (0.32)	0.29 (0.57)	0.9927
**Red meat**	0.20 (0.44)	0.41 (0.35)	0.01 (0.15)	<0.0001
**Nuts and legumes**	0.71 (0.92)	0.62 (0.83)	0.85 (0.97)	<0.0001
**Full-fat dairy**	-	1.05 (1.11)	-	-
**Fish/fish oil**	-	0.08 (0.15)	-	-
**Metabolite concentration, median (IQR)**				
**Proline betaine**	1.81 (3.82)	2.33 (5.52)	1.40 (2.47)	<0.0001
**Hippuric acid**	10.01 (9.87)	9.68 (9.03)	10.07 (10.36)	0.8848
**TMAO**	2.53 (1.95)	2.68 (1.96)	2.24 (1.99)	<0.0001
**3-methylhistidine**	7.17 (4.12)	8.64 (4.90)	6.14 (2.24)	<0.0001
**Carnitine**	15.61 (3.82)	15.35 (3.69)	15.89 (3.98)	0.0117
**Tryptophan betaine**	1.27 (0.37)	1.19 (0.14)	1.47 (0.37)	<0.0001
**Fatty acids, median (IQR) *^d^***				
**Myristic acid (14:0)**	-	2.19 (0.74)	-	-
**Pentadecanoic acid (15:0)**	-	0.24 (0.08)	-	-
**Heptadecanoic acid (17:0)**	-	0.69 (0.23)	-	-
**Eicosapentaenoic acid (EPA or 20:5n-3)**	-	0.51 (0.26)	-	-
**Docosahexaenoic acid (DHA or 22:6n-3)**	-	0.67 (0.29)	-	-

FFQ = Food frequency questionnaire; TMAO = trimethylamine *N*-oxide Wilcoxon’s rank sum test was used to compare continuous variables, and chi-square was used to compare categorical variables by cohort. *^a^* GDM was defined based on the Born in Bradford oral glucose tolerance test criteria, self-reported GDM, and insulin use in pregnancy in the START cohort, whereas the International Association of the Diabetes and Pregnancy Study Groups criteria (75 g OGTT with fasting glucose ≥ 5.1 mmol/L, 1 h ≥ 10.0 mmol/L, 2 h ≥ 8.5 mmol/L) was used in the FAMILY cohort. *^b^* The maximum social disadvantage index was five and the lowest possible score was zero, reflecting the least social disadvantage. *^c^* FFQ was implemented within a one-year time period of the blood draw. *^d^* Fatty acids data were only available in the FAMILY cohort.

**Table 2:** Results from random effects hierarchical modelling examining the association of dietary and non-dietary factors with food-based metabolites.

	Proline Betaine	Hippuric Acid	3-Methyl Histidine	Carnitine	Tryptophan Betaine	TMAO
Factor	b (95% CI)	b (95% CI)	b (95% CI)	b (95% CI)	b (95% CI)	b (95% CI)
**Age (years)**	0.04 * (0.01, 0.07)	0.01 (0.00, 0.03)	0.00 (−0.01, 0.01)	0.00 (0.00, 0.00)	0.00 (0.00, 0.03)	0.02 * (0.00, 0.04)
**Gestational age (weeks)**	0.02 (−0.03, 0.06)	0.01 (−0.01, 0.03)	0.01 * (0.00, 0.02)	−0.01 *** (−0.02, −0.01)	0.00 (0.00, 0.00)	0.01 (−0.01, 0.03)
**Parity**	−0.10 (−0.25, 0.06)	0.03 (−0.05, 0.11)	−0.01 (−0.05, 0.03)	0.01 (−0.01, 0.02)	−0.01 (−0.02, 0.01)	0.01 (−0.07, 0.09)
**Gestational diabetes (GDM)**	0.05 (−0.24, 0.35)	0.06 (−0.10, 0.21)	0.02 (−0.05, 0.10)	0.02 (−0.02, 0.05)	0.02 (−0.01, 0.05)	0.03 (−0.13, 0.19)
**Pre-pregnancy BMI (kg/m^2^)**	−0.02 (−0.05, 0.00)	−0.01 (−0.02, 0.00)	−0.01 (−0.01, 0.00)	0.00 (0.00, 0.00)	0.00 (0.00, 0.00)	−0.01 (−0.02, 0.01)
**Smoking history** **(ever vs. never smoked)**	−0.60 *** (−0.95, −0.25)	−0.12 (−0.30, 0.06)	0.04 (−0.06, 0.13)	0.06 ** (0.02, 0.10)	0.00 (−0.03, 0.03)	−0.01 (−0.20, 0.17)
**Physical activity (low vs. high)**	−0.13 (−0.42, 0.17)	0.02 (−0.14, 0.18)	−0.03 (−0.10, 0.05)	−0.01 (−0.04, 0.02)	0.00 (−0.03, 0.03)	−0.04 (−0.21, 0.12)
**Social disadvantage index**	−0.05 (−0.15, 0.06)	−0.02 (−0.08, 0.03)	0.00 (−0.03, 0.02)	0.00 (−0.01, 0.01)	0.00 (−0.01, 0.01)	−0.01 (−0.06, 0.05)
**Fiber intake (g/day)**	0.01 (−0.01, 0.02)	0.01 (−0.01, 0.02)	0.00 (−0.01, 0.00)	0.00 (0.00, 0.00)	2.68 × 10^−3^ ** (0.00, 0.00)	0.00 (−0.01, 0.01)
**Energy intake (kcal)**	0.00 (0.00, 0.00)	−1.6 × 10^−4^ ** (−0.00, −0.00)	0.00 (0.00, 0.00)	0.00 (0.00, 0.00)	−3 × 10^−5^ * (0.00, 0.00)	0.00 (0.00, 0.00)
**FFQ before blood draw vs. FFQ at the same time as blood draw**	0.02 (−0.30, 0.35)	0.11 (−0.05, 0.27)	−0.05 (−0.13, 0.04)	0.00 (−0.03, 0.04)	−0.01 (−0.04, 0.02)	0.09 (−0.08, 0.26)
**FFQ after blood draw vs. FFQ at the same time as blood draw**	0.50 (−0.34, 1.34)	0.08 (−0.37, 0.54)	0.04 (−0.18, 0.26)	0.06 (−0.04, 0.16)	−0.04 (−0.12, 0.04)	−0.11 (−0.56, 0.35)
**Citrus food** **(servings/day)**	0.27 *** (0.20, 0.34)					
**Fruits and vegetables** **(servings/day)**		0.22 ** (0.08, 0.36)				
**Tea** **(servings/day)**		0.01 (−0.01, 0.04)				
**Coffee** **(servings/day)**		0.02 (0.00, 0.04)				
**Chicken** **(servings/day)**			0.02 * (0.00, 0.04)			
**Red meat** **(servings/day)**			0.03 * (0.01, 0.06)	0.00 (0.00, 0.01)		0.00 (−0.04, 0.04)
**Eggs** **(servings/day)**			0.01 (−0.01, 0.02)			0.00 (−0.03, 0.04)
**Nuts and legumes** **(servings/day)**			0.02 (−0.02, 0.06)		0.02 * (0.00, 0.03)	
**Canned fish** **(servings/day)**						0.01 (−0.03, 0.04)
**Fried fish** **(servings/day)**						0.01 (−0.03, 0.05)
**Seafood** **(servings/day)**						0.08 *** (0.04, 0.12)

* *p* ≤ 0.05, ** *p* ≤ 0.01, *** *p* ≤ 0.001. FFQ = Food frequency questionnaire; TMAO = trimethylamine *N*-oxide.

**Table 3:** Results from ordinary least squares regression examining the association of dietary and non-dietary factors with serum non-esterified fatty acid (NEFA) in the FAMILY cohort.

	Even-Chain SFA	Odd-Chain SFA		ω-3 PUFA		
	14:0	15:0	17:0	EPA	DHA	EPA + DHA
**Variable**	**b (95% CI)**	**b (95% CI)**	**b (95% CI)**	**b (95% CI)**	**b (95% CI)**	**b (95% CI)**
**Age (years)**	4.24 × 10^−3^ (−0.00, 0.01)	−3.54 × 10^−4^ (−0.01, 0.01)	−0.01 (−0.01, 0.00)	−0.01 (−0.03, 0.00)	−4.77 × 10^−3^ (−0.01, 0.00)	−0.01 (−0.02, 0.00)
**Gestational age (weeks)**	−0.01 (−0.02, 0.00)	−0.02 *** (−0.03, −0.01)	−0.01 ** (−0.02, −0.00)	−0.01 (−0.03, 0.01)	−0.01 (−0.02, 0.00)	−0.01 (−0.02, 0.00)
**Parity**	−0.01 (−0.04, 0.03)	2.105 × 10−4 (−0.03, 0.03)	2.21 × 10^−3^ (−0.03, 0.03)	−2.07 × 10^−5^ (−0.06, 0.06)	−0.03 (−0.08, 0.01)	−0.02 (−0.07, 0.03)
**Gestational diabetes (GDM)**	0.02 (−0.07, 0.10)	−0.01 (−0.09, 0.07)	−0.06 (−0.14, 0.03)	−0.06 (−0.22, 0.10)	−0.07 (−0.18, 0.04)	−0.07 (−0.19, 0.05)
**Pre-pregnancy BMI (kg/m^2^)**	−0.01 * (−0.01, −0.00)	−0.01 ** (−0.01, −0.00)	−0.01 * (−0.01, −0.00)	−2.86 × 10^−3^ (−0.01, 0.01)	−0.01 * (−0.02, −0.00)	−0.01 (−0.01, 0.00)
**Smoking history** **(ever vs. never smoked)**	−0.02 (−0.08, 0.05)	−0.04 (−0.10, 0.03)	−0.05 (−0.12, 0.01)	−0.01 (−0.13, 0.10)	−0.05 (−0.14, 0.03)	−0.04 (−0.12, 0.05)
**Physical activity** **(low vs. high)**	−0.01 (−0.09, 0.08)	−0.01 (−0.09, 0.06)	−0.10 ** (−0.17, −0.02)	−0.03 (−0.18, 0.11)	−0.03 (−0.12, 0.06)	−0.03 (−0.13, 0.08)
**Social disadvantage index**	−0.02 (−0.04, 0.01)	−1.79 × 10^−3^ (−0.03, 0.03)	0.02 (−0.01, 0.05)	0.04 (−0.03, 0.10)	0.04 (−0.00, 0.08)	0.04 (−0.01, 0.08)
**Fiber intake** **(g/day)**	−1.12 × 10^−3^ (−0.01, 0.01)	2.84 × 10^−3^ (−0.00, 0.01)	1.45 × 10^−3^ (−0.00, 0.01)	4.51 × 10^−3^ (−0.00, 0.01)	2.01 × 10^−3^ (−0.00, 0.01)	3.48 × 10^−3^ (−0.00, 0.01)
**Energy intake (kcal)**	−1.05 × 10^−5^ (−0.00, −0.00)	−4.76 × 10^−5^ (−0.00, 0.00)	−2.42 × 10^−5^ (−0.00, 0.00)	−8.23 × 10^−5^ (−0.00, 0.00)	−4.77 × 10^−5^ (−0.00, 0.00)	−6.21 × 10^−5^ (−0.00, 0.00)
**FFQ before blood draw vs. FFQ at the same time as blood draw**	−0.03 (−0.10, 0.04)	0.06 (−0.01, 0.13)	0.01 (−0.06, 0.08)	−3.01 × 10^−3^ (−0.14, 0.14)	0.05 (−0.04, 0.15)	0.02 (−0.07, 0.12)
**FFQ after blood draw vs. FFQ at the same time as blood draw**	−0.05 (−0.26, 0.16)	0.02 (−0.09, 0.13)	0.04 (−0.10, 0.19)	0.06 (−0.26, 0.38)	0.24 * (0.02, 0.46)	0.16 (−0.07, 0.40)
**Full-fat dairy** **(servings/day)**	0.02 (−0.02, 0.06)	0.06 *** (0.03, 0.10)	0.04 ** (0.01, 0.07)			
**Fish/Fish oil** **(servings/day)**				0.05 (−0.00, 0.11)	0.11 *** (0.07, 0.14)	0.08 *** (0.04, 0.12)

* *p* ≤ 0.05, ** *p* ≤ 0.01, *** *p* ≤ 0.001.

## Data Availability

Data described in the manuscript, code book, and analytic code will not be made available because participants in the FAMILY and START studies did not consent to public sharing of their data at the time of recruitment. Datasets can be made available from the corresponding author on reasonable request.

## Supplementary Materials

The following supporting information can be downloaded at: https://www.mdpi.com/article/10.3390/nu14122503/s1, Table S1: Results from ordinary least squares regression examining the association of dietary and non-dietary factors with food-based metabolites; Table S2: Results of model fitting analyses examining the association of dietary and non-dietary factors with food metabolites; Table S3: Results from ordinary least squares regression examining the association of dietary and non-dietary factors with food-based metabolites in FAMILY cohort; Table S4: Results from ordinary least squares regression examining the association of dietary and non-dietary factors with food-based metabolites in START cohort; Table S5: Results from ordinary least squares regression examining the association of dietary and non-dietary factors with serum non-esterified fatty acid (NEFA) in FAMILY cohort; Figure S1. Consort flow diagram outlining selection criteria used in a cross-sectional study involving participants from the FAMILY and START birth cohorts; Figure S2A: Weighted R_partial_^2^ for each factor showing the percentage of explained variability in Proline betaine in FAMILY cohort; Figure S2B: Weighted R_partial_^2^ for each factor showing the percentage of explained variability in Proline betaine in START cohort; Figure S3A: Weighted R_partial_^2^ for each factor showing the percentage of explained variability in Hippuric acid in FAMILY cohort; Figure S3B: Weighted R_partial_^2^ for each factor showing the percentage of explained variability in Hippuric acid in START cohort; Figure S4A: Weighted R_partial_^2^ for each factor showing the percentage of explained variability in Tryptophan betaine in FAMILY cohort; Figure S4B: Weighted R_partial_^2^ for each factor showing the percentage of explained variability in Tryptophan betaine in START cohort; Figure S5A: Weighted R_partial_^2^ for each factor showing the percentage of explained variability in Carnitine in FAMILY cohort; Figure S5B: Weighted R_partial_^2^ for each factor showing the percentage of explained variability in Carnitine in START cohort; Figure S6A: Weighted R_partial_^2^ for each factor showing the percentage of explained variability in trimethylamine *N*-oxide (TMAO) in FAMILY cohort; Figure S6B: Weighted R_partial_^2^ for each factor showing the percentage of explained variability in trimethylamine *N*-oxide (TMAO) in START cohort; Figure S7A: Weighted R_partial_^2^ for each factor showing the percentage of explained variability in 3-methylhistidine in FAMILY cohort; Figure S7B: Weighted R_partial_^2^ for each factor showing the percentage of explained variability in 3-methylhistidine in START cohort; Figure S8: Weighted R_partial_^2^ for each factor showing the percentage of explained variability in Eicosapentaenoic acid (EPA, 20:5n-3) in FAMILY cohort; Figure S9: Weighted R_partial_^2^ for each factor showing the percentage of explained variability in Docosahexaenoic acid (DHA; 22:6n-3) in FAMILY cohort; Figure S10: Weighted R_partial_^2^ for each factor showing the percentage of explained variability in EPA + DHA in FAMILY cohort; Figure S11: Venn diagram showing overlap of serum metabolites based on the cluster effect by ethnicity/cohort.
