# Supplementary material for: Sources of Variation in Food-Related Metabolites during Pregnancy
Source: Nutrients. 2022 Jun 16;14(12):2503. doi: 10.3390/nu14122503 (PMC9227758; doi:10.3390/nu14122503)
Supplement: Supplementary file 1 [file nutrients-14-02503-s001.zip › nutrients-1756631-supplementary.pdf]

Table S1: Results from ordinary least squares regression examining the association of dietary and non-dietary factors with food-based metabolites

	**Hippuric acid**	**Carnitine**
**Variable**	**b (95% CI)**	**b (95% CI)**
**Age (years)**	0.01<br>(-0.01, 0.03)	0.00<br>(-0.00, 0.00)
**Gestational age (weeks)**	0.01<br>(-0.01, 0.03)	-0.01**<br>(-0.02, -0.01)
**Parity**	0.03<br>(-0.05, 0.11)	0.00<br>(-0.01, 0.02)
**Gestational diabetes (GDM)**	0.06<br>(-0.10, 0.23)	0.02<br>(-0.02, 0.05)
**Pre-pregnancy BMI (kg/m^2)**	-0.01<br>(-0.03, 0.00)	0.00<br>(-0.00, 0.00)
**Smoking history<br>(ever vs. never smoked)**	-0.13<br>(-0.29, 0.03)	0.06*<br>(0.02, 0.10)
**Physical activity<br>(low vs. high)**	0.02<br>(-0.11, 0.15)	-0.01<br>(-0.04, 0.02)
**Social disadvantage index**	-0.02<br>(-0.08, 0.04)	0.00<br>(-0.01, 0.01)
**Fiber intake<br>(g/day)**	0.01<br>(-0.00, 0.02)	0.00<br>(-0.00, 0.00)
**Energy intake (kcal)**	0.00*<br>(-0.00, -0.00)	0.00<br>(-0.00, 0.00)
**FFQ before blood draw<br>vs. FFQ at the same time as blood draw**	0.10<br>(-0.07, 0.26)	0.01<br>(-0.03, 0.05)
**FFQ after blood draw<br>vs. FFQ at the same time as blood draw**	0.08<br>(-0.18, 0.34)	0.06<br>(-0.07, 0.19)
**Ethnicity (White European vs. South Asian)**	0.04<br>(-0.18, 0.26)	-0.02<br>(-0.07, 0.04)
**Fruits and vegetables<br>(servings/day)**	0.22*<br>(0.08, 0.36)	
**Tea<br>(servings/day)**	0.01<br>(-0.01, 0.04)	
**Coffee<br>(servings/day)**	0.02<br>(-0.00, 0.04)	
**Red meat<br>(servings/day)**		0.00<br>(-0.00, 0.01)

*p ≤ 0.01, **p ≤ 0.001

Table S2: Results of model fitting analyses examining the association of dietary and non-dietary factors with food metabolites

	−2 Log L	BIC	AIC	S_b^2	S_w^2
**Proline Betaine**					
**Model 1**	–	–	–	0.14	0.13
**Model 2**	2100.0	2101.4	2104.0	0.02	0.11
**Model 3**	1779.0	1780.4	1783.0	0.20	0.12
**Hippuric acid**					
**Model 1**	–	–	–	0.00	0.03
**Model 2**	1394.7	1395.3	1396.7	0.00	0.03
**Model 3**	1182.1	1182.8	1184.1	0.00	0.03
**3-methylhistidine**					
**Model 1**	–	–	–	0.07	0.01
**Model 2**	515.3	518.8	525.3	0.02	0.01
**Model 3**	496.0	497.4	500.0	0.02	0.01
**Carnitine**					
**Model 1**	–	–	–	0.00	0.00
**Model 2**	-387.0	-385.6	-383.0	0.00	0.00
**Model 3**	-298.6	-297.9	-296.6	0.00	0.00
**Tryptophan betaine**					
**Model 1**	–	–	–	0.03	0.00
**Model 2**	-588.1	-586.7	-584.1	0.02	0.00
**Model 3**	-489.4	-488.0	-485.4	0.02	0.00
**TMAO**					
**Model 1**	–	–	–	0.07	0.04
**Model 2**	1421.5	1422.9	1425.5	0.02	0.03
**Model 3**	1211.1	1211.8	1213.1	0.00	0.04

AIC = Akaike Information Criterion, BIC = Bayesian Information Criterion, -2 Log L = -2 log likelihood, S_b^2 = Sum of square between, S_w^2 = Sum of square within
Model 1: Unconditional (intercept only) model
Model 2: Random Intercept with Fixed Level-1 Factors (dietary factors)
Model 3: Random Intercept with Fixed Level-1 Factors (dietary and non-dietary factors)

Table S3: Results from ordinary least squares regression examining the association of dietary and non-dietary factors with food-based metabolites in FAMILY cohort

	**Proline betaine**	**Hippuric acid**	**3-Methyl histidine**	**Carnitine**	**Tryptophan betaine**	**TMAO**
**Variable**	**b (95% CI)**	**b (95% CI)**	**b (95% CI)**	**b (95% CI)**	**b (95% CI)**	**b (95% CI)**
**Age (years)**	0.06** (0.02, 0.10)	0.02 (-0.01, 0.04)	0.00 (-0.01, 0.02)	0.00 (-0.00, 0.01)	0.00 (-0.00, 0.00)	0.02 (-0.01, 0.04)
**Gestational age (weeks)**	0.02 (-0.02, 0.10)	0.00 (-0.02, 0.02)	0.01 (-0.01, 0.02)	-0.01*** (-0.02, 0.01)	0.00 (-0.00, 0.00)	0.01 (-0.01, 0.04)
**Parity**	-0.06 (-0.26, 0.15)	-0.02 (-0.11, 0.08)	0.00 (-0.07, 0.07)	-0.01 (-0.03, 0.01)	-0.01 (-0.02, 0.00)	-0.04 (-0.12, 0.05)
**Gestational diabetes (GDM)**	-0.15 (-0.65, 0.35)	0.01 (-0.23, 0.24)	0.06 (-0.07, 0.19)	0.03 (-0.02, 0.08)	-0.01 (-0.04, 0.02)	-0.07 (-0.29, 0.15)
**Pre-pregnancy BMI (kg/m^2)**	-0.03* (-0.06, -0.00)	-0.01 (-0.03, 0.01)	-0.00 (-0.01, 0.00)	-0.00 (-0.01, 0.00)	0.00 (-0.00, 0.00)	-0.01 (-0.02, 0.01)
**Smoking history (ever vs. never smoked)**	-0.53** (-0.91, 0.14)	-0.10 (-0.26, 0.07)	0.03 (-0.07, 0.13)	0.06** (0.02, 0.10)	0.00 (-0.02, 0.00)	0.01 (-0.17, 0.19)
**Physical activity (low vs. high)**	0.06 (-0.31, 0.43)	-0.02 (-0.18, 0.14)	-0.05 (-0.15, 0.06)	-0.02 (-0.06, 0.03)	0.00 (-0.03, 0.02)	-0.02 (-0.21, 0.18)
**Social disadvantage index**	-0.03 (-0.22, 0.17)	-0.01 (-0.10, 0.07)	0.00 (-0.04, 0.05)	-0.00 (-0.02, 0.01)	0.00 (-0.01, 0.01)	0.04 (-0.03, 0.11)
**Fiber intake (g/day)**	-0.00 (-0.03, 0.02)	0.01 (-0.00, 0.02)	-0.00 (-0.01, 0.01)	0.00 (-0.00, 0.00)	0.00 (-0.00, 0.00)	0.00 (-0.01, 0.02)
**Energy intake (kcal)**	-0.00 (-0.00, 0.00)	-0.00* (-0.00, -0.00)	-0.00 (0.00, 0.00)	-0.00 (-0.00, 0.00)	0.00 (-0.00, 0.00)	-0.00 (-0.00, 0.00)
**FFQ before blood draw vs. FFQ at the same time as blood draw**	0.06 (-0.29, 0.40)	0.09 (-0.09, 0.26)	-0.06 (-0.16, 0.05)	0.02 (-0.02, 0.06)	-0.01 (-0.03, 0.02)	0.10 (-0.08, 0.28)
**FFQ after blood draw vs. FFQ at the same time as blood draw**	0.08 (-0.70, 0.86)	-0.02 (-0.46, 0.41)	0.10 (-0.30, 0.50)	0.05 (-0.10, 0.21)	-0.02 (-0.11, 0.06)	-0.14 (-0.72, 0.45)

**Citrus food (servings/day)**	0.40*** (0.24, 0.57)					
**Fruits and vegetables (servings/day)**		0.30*** (0.16, 0.45)				
**Tea (servings/day)**		0.00 (-0.02, 0.03)				
**Coffee (servings/day)**		0.01 (-0.01, 0.04)				
**Chicken (servings/day)**			0.03 (-0.00, 0.06)			
**Red meat (servings/day)**			0.01 (-0.03, 0.05)	0.02* (0.00, 0.04)		0.04 (-0.02, 0.10)
**Eggs (servings/day)**			0.03 (-0.00, 0.06)			0.03 (-0.04, 0.10)
**Nuts and legumes (servings/day)**			0.06* (0.00, 0.12)		0.02* (0.00, 0.04)	
**Canned fish (servings/day)**						0.02 (-0.01, 0.06)
**Fried fish (servings/day)**						0.02 (-0.02, 0.07)
**Seafood (servings/day)**						0.07** (0.02, 0.11)

*$p \leq 0.05$, **$p \leq 0.01$, ***$p \leq 0.001$

Table S4: Results from ordinary least squares regression examining the association of dietary and non-dietary factors with food-based metabolites in START cohort

	**Proline betaine**	**Hippuric acid**	**3-Methyl histidine**	**Carnitine**	**Tryptophan betaine**	**TMAO**
**Variable**	**b (95% CI)**	**b (95% CI)**	**b (95% CI)**	**b (95% CI)**	**b (95% CI)**	**b (95% CI)**
**Age (years)**	-0.01 (-0.07, 0.04)	-0.01 (-0.04, 0.02)	0.00 (-0.01, 0.01)	-0.00 (-0.01, 0.00)	0.00 (-0.01, 0.01)	0.03 (-0.01, 0.06)
**Gestational age (weeks)**	0.02 (-0.08, 0.13)	0.05 (-0.01, 0.11)	-0.00 (-0.03, 0.02)	-0.02*** (-0.03, -0.01)	-0.01 (-0.02, 0.00)	0.02 (-0.03, 0.07)
**Parity**	-0.18 (-0.45, 0.10)	0.11 (-0.02, 0.24)	-0.03 (-0.09, 0.04)	0.03 (-0.00, 0.06)	-0.01 (-0.04, 0.03)	0.07 (-0.06, 0.21)
**Gestational diabetes (GDM)**	0.18 (-0.18, 0.55)	0.09 (-0.14, 0.32)	-0.02 (-0.11, 0.07)	0.01 (-0.03, 0.06)	0.04 (-0.00, 0.09)	0.05 (-0.18, 0.27)
**Pre-pregnancy BMI (kg/m^2)**	0.01 (-0.04, 0.05)	-0.02 (-0.05, 0.00)	-0.01 (-0.02, 0.00)	0.00 (-0.00, 0.01)	0.00 (-0.00, 0.01)	-0.02 (-0.04, 0.01)
**Physical activity (low vs. high)**	-0.57* (-0.12, 0.03)	-0.05 (-0.27, 0.17)	-0.01 (-0.11, 0.09)	-0.00 (-0.05, 0.05)	0.03 (-0.02, 0.08)	-0.04 (-0.36, 0.28)
**Social disadvantage index**	-0.05 (-0.18, 0.09)	-0.03 (-0.10, 0.05)	-0.00 (-0.04, 0.03)	0.01 (-0.01, 0.02)	-0.00 (-0.02, 0.02)	-0.07 (-0.15, 0.01)
**Fiber intake (g/day)**	0.01 (-0.02, 0.04)	0.01 (-0.01, 0.03)	-0.00 (-0.01, 0.00)	-0.00 (-0.01, 0.00)	0.00** (0.00, 0.01)	-0.00 (-0.02, 0.01)
**Energy intake (kcal)**	-0.00 (-0.00, 0.00)	0.00* (0.00, 0.00)	0.00 (-0.00, 0.00)	0.00** (0.00, 0.00)	-0.00* (-0.00, -0.00)	0.00 (-0.00, 0.00)
**FFQ before blood draw vs. FFQ at the same time as blood draw**	-0.76 (-1.58, 0.07)	0.24 (-0.25, 0.72)	0.00 (-0.18, 0.19)	0.05 (-0.14, 0.05)	-0.01 (-0.08, 0.05)	0.02 (-0.37, 0.41)
**FFQ after blood draw vs. FFQ at the same time as blood draw**	0.94** (0.29, 1.59)	0.14 (-0.23, 0.50)	-0.07 (-0.28, 0.13)	0.06 (-0.14, 0.27)	-0.06 (-0.24, 0.12)	-0.19 (-0.64, 0.26)
**Citrus food (servings/day)**	0.18*** (0.17, 0.28)					

**Fruits and vegetables**<br>**(servings/day)**		0.11<br>(-0.09, 0.30)				
**Tea**<br>**(servings/day)**		0.03<br>(-0.02, 0.08)				
**Coffee**<br>**(servings/day)**		0.02<br>(-0.04, 0.07)				
**Chicken**<br>**(servings/day)**			-0.00<br>(-0.03, 0.03)			
**Red meat**<br>**(servings/day)**			0.06**<br>(0.02, 0.10)	-0.00<br>(-0.01, 0.01)		0.00<br>(-0.06, 0.06)
**Eggs**<br>**(servings/day)**			-0.01<br>(-0.02, 0.01)			0.00<br>(-0.03, 0.07)
**Nuts and legumes**<br>**(servings/day)**			-0.05<br>(-0.11, 0.01)		0.02<br>(-0.01, 0.05)	
**Canned fish**<br>**(servings/day)**						-0.08<br>(-0.24, 0.07)
**Fried fish**<br>**(servings/day)**						-0.00<br>(-0.08, 0.07)
**Seafood**<br>**(servings/day)**						0.11*<br>(0.01, 0.21)

*$p \leq 0.05$, **$p \leq 0.01$, ***$p \leq 0.001$

Table S5: Results from ordinary least squares regression examining the association of dietary and non-dietary factors with serum non-esterified fatty acid (NEFA) in FAMILY cohort

	**ω-3 PUFA**		
	**EPA**	**DHA**	**EPA + DHA**
**Variable**	**b (95% CI)**	**b (95% CI)**	**b (95% CI)**
**Age (years)**	-0.01 (-0.02, 0.00)	-2.92×10^{-3} (-0.01, 0.01)	-0.01 (-0.02, 0.00)
**Gestational age (weeks)**	-0.01 (-0.03, 0.00)	-0.01* (-0.02, -0.00)	-0.01* (-0.02, -0.00)
**Parity**	-0.01 (-0.07, 0.06)	-0.05* (-0.09, -0.00)	-0.03 (-0.08, 0.02)
**Gestational diabetes (GDM)**	-0.06 (-0.22, 0.10)	-0.06 (-0.17, 0.05)	-0.07 (-0.18, 0.05)
**Pre-pregnancy BMI (kg/m^2)**	-3.34×10^{-3} (-0.01, 0.01)	-0.01** (-0.02, -0.00)	-0.01 (-0.01, 0.00)
**Smoking history (ever vs. never smoked)**	-0.01 (-0.13, 0.11)	-0.04 (-0.13, 0.04)	-0.03 (-0.12, 0.05)
**Physical activity (low vs. high)**	-0.03 (-0.18, 0.12)	-0.02 (-0.12, 0.08)	-0.02 (-0.13, 0.09)
**Social disadvantage index**	0.03 (-0.04, 0.10)	0.03 (-0.01, 0.07)	0.03 (-0.02, 0.08)
**Fiber intake (g/day)**	0.01 (-0.00, 0.01)	3.71×10^{-3} (-0.00, 0.01)	4.88×10^{-3} (-0.00, 0.01)
**Energy intake (kcal)**	-7.71×10^{-5} (-0.00, 0.00)	-3.76×10^{-5} (-0.00, 0.00)	-5.39×10^{-5} (-0.00, 0.00)
**FFQ before blood draw vs. FFQ at the same time as blood draw**	-0.01 (-0.15, 0.13)	0.04 (-0.05, 0.13)	0.02 (-0.08, 0.11)
**FFQ after blood draw vs. FFQ at the same time as blood draw**	0.06 (-0.26, 0.39)	0.25 (-0.04, 0.54)	0.17 (-0.11, 0.44)
**Fish (servings/day)**	0.02 (-0.01, 0.05)	0.04** (0.01, 0.06)	0.03* (0.01, 0.05)

*$p \leq 0.05$, **$p \leq 0.01$

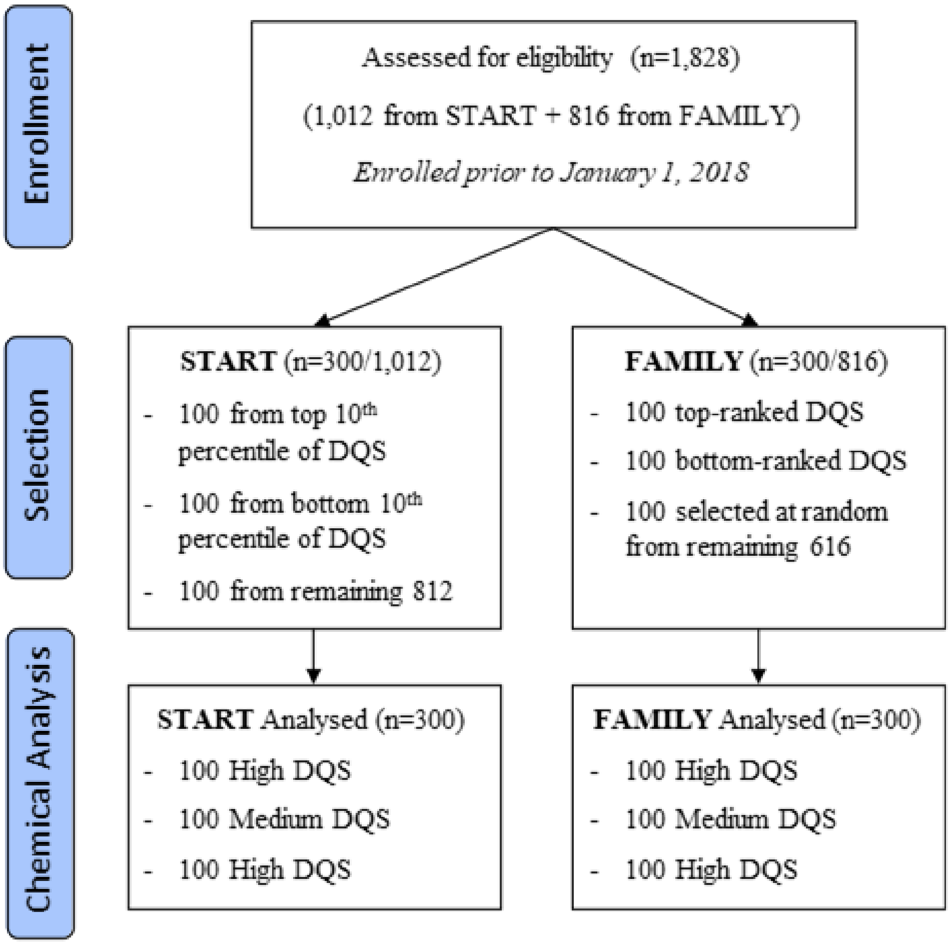

Figure S1. Consort flow diagram outlining selection criteria used in a cross-sectional study involving participants from the FAMILY and START birth cohorts

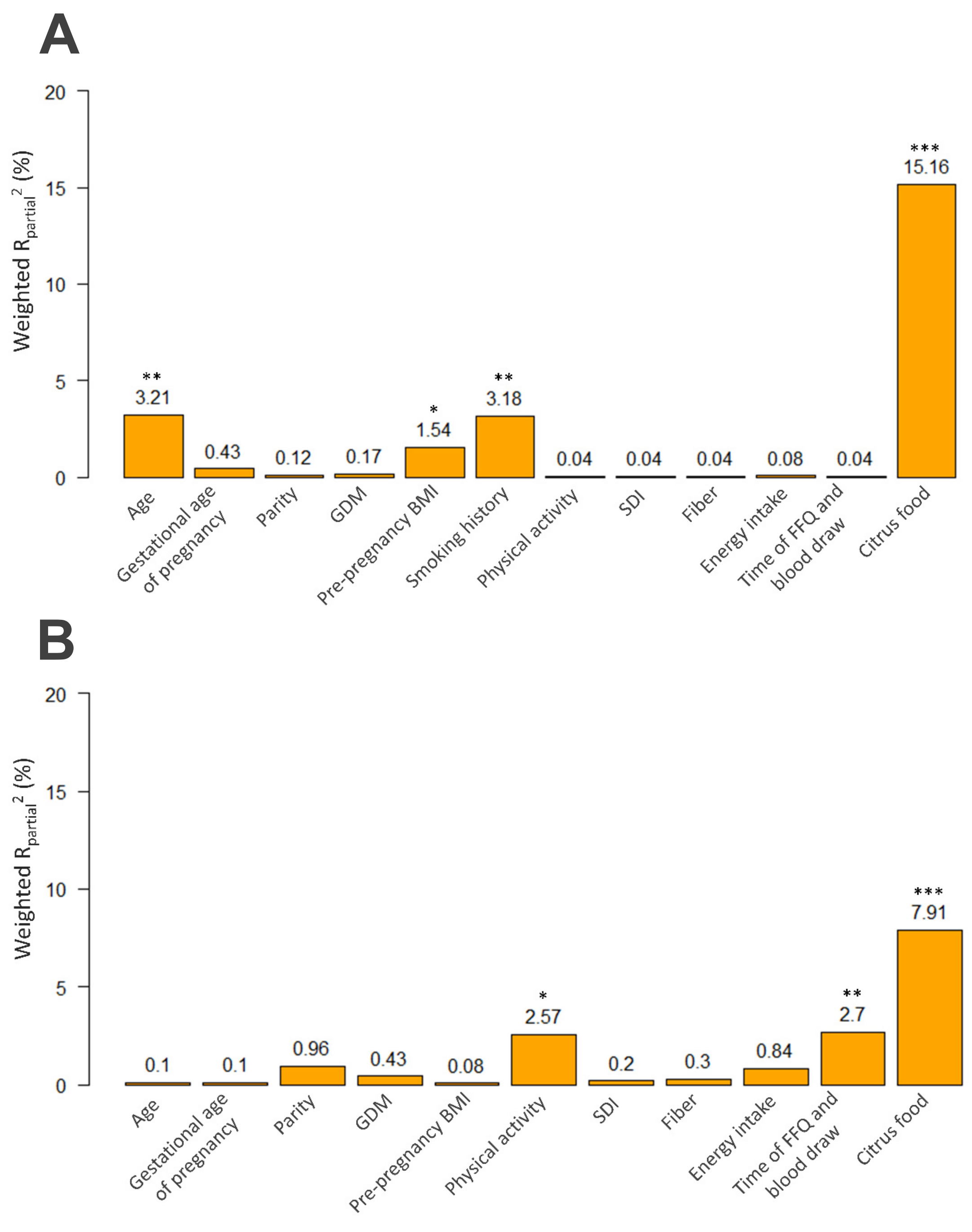

Figure S2: Weighted $R_{partial}^2$ for each factor showing the percentage of explained variability in Proline betaine in (A) FAMILY and (B) START cohort

Statistical significance was based on hierarchical linear models. * $p \leq 0.05$, **$p \leq 0.01$, ***$p \leq 0.001$

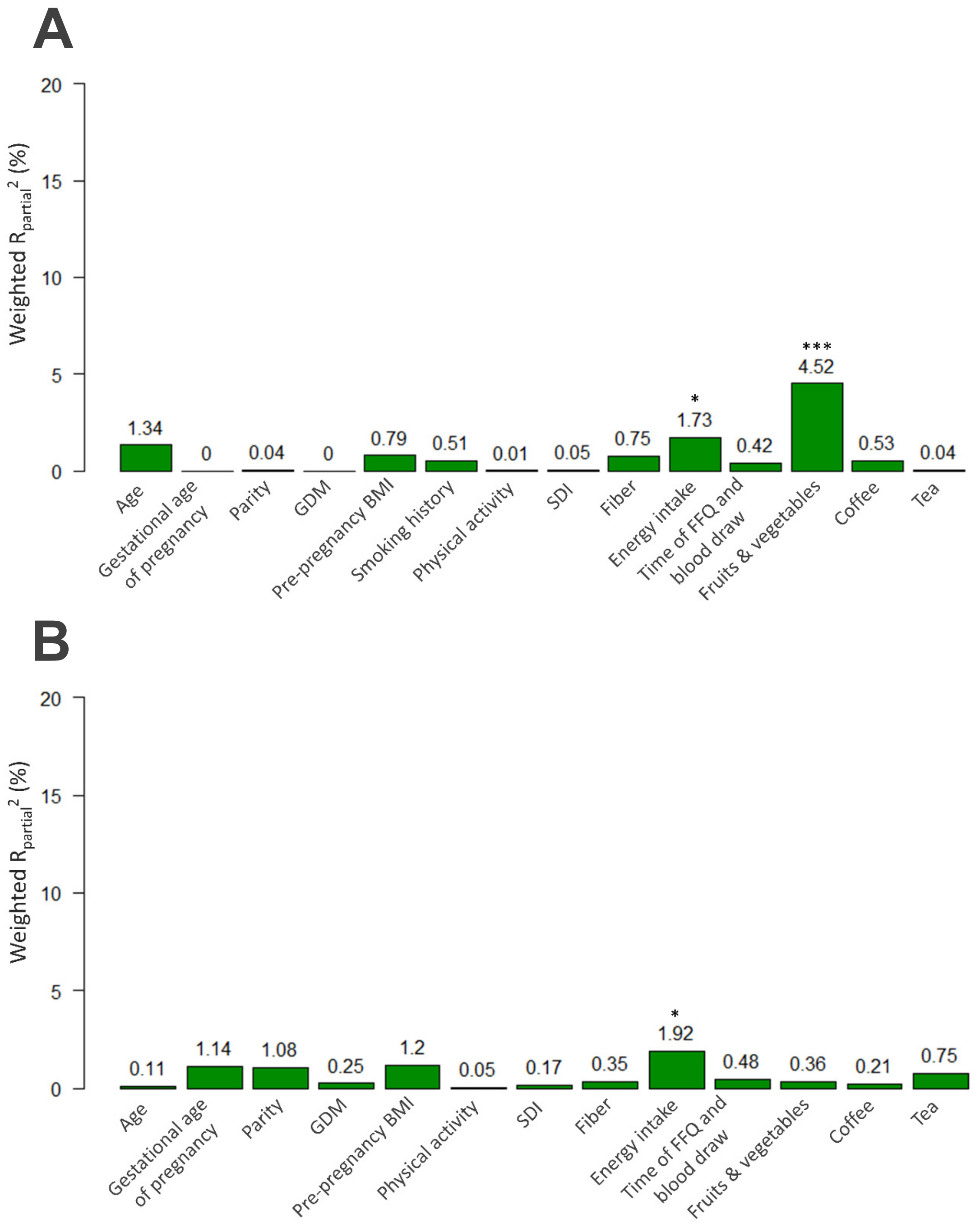

Figure S3: Weighted $R_{partial}^2$ for each factor showing the percentage of explained variability in Hippuric acid in (A) FAMILY and (B) START cohort

Statistical significance was based on hierarchical linear models. * $p \leq 0.05$, ***$p \leq 0.001$

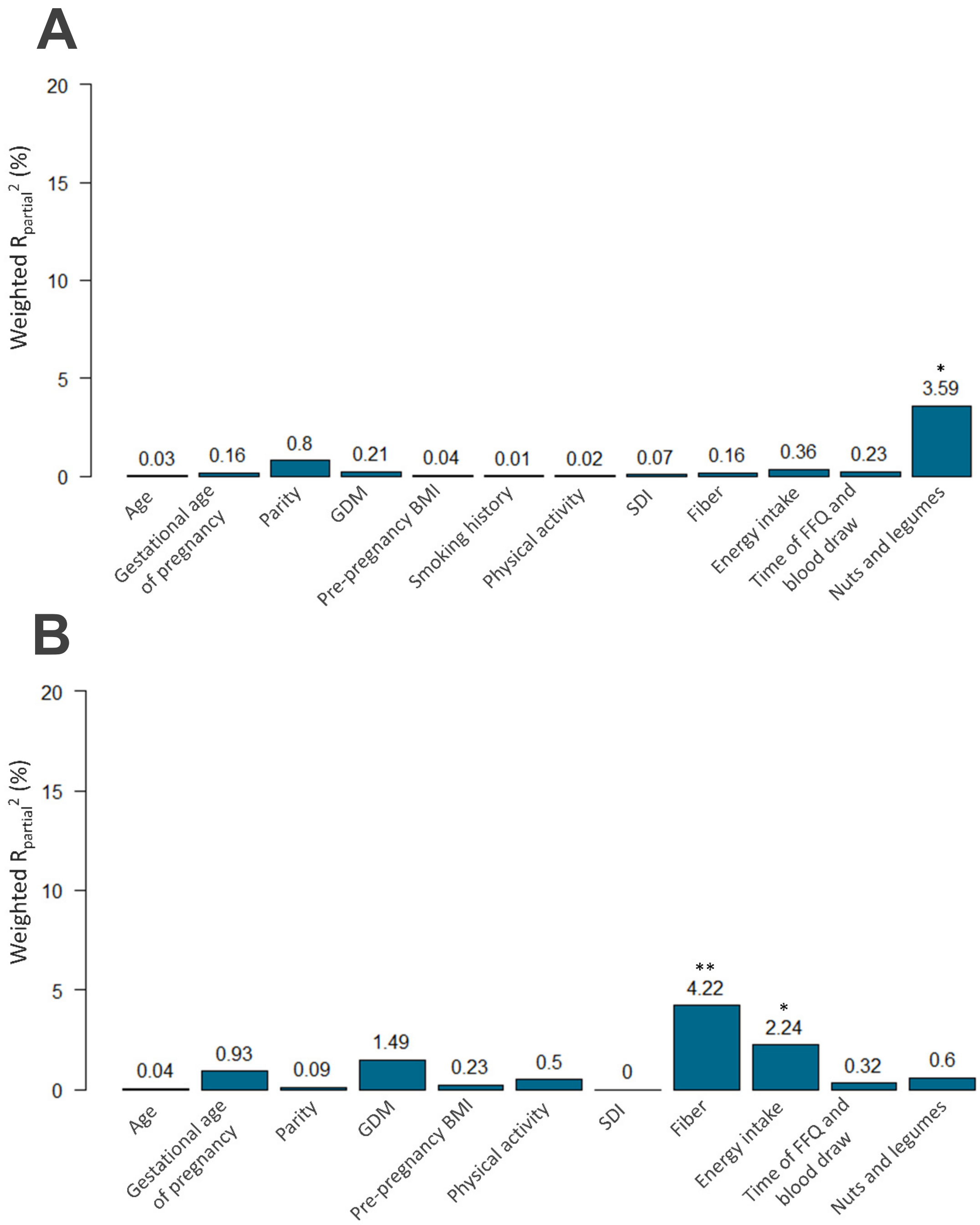

Figure S4: Weighted $R_{partial}^2$ for each factor showing the percentage of explained variability in Tryptophan betaine in (A) FAMILY and (B) START cohort

Statistical significance was based on hierarchical linear models. * $p \leq 0.05$, **$p \leq 0.01$

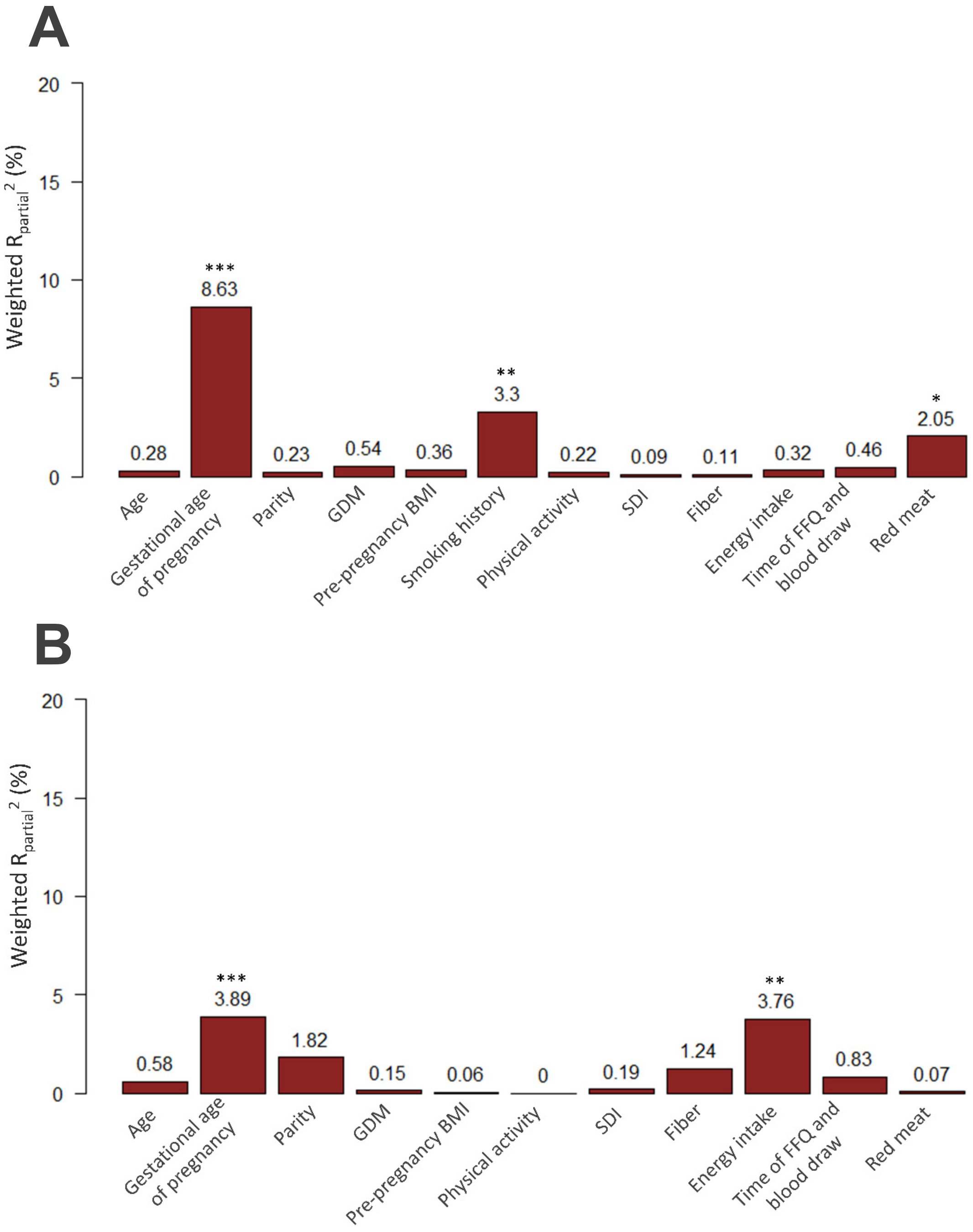

Figure S5: Weighted $R_{partial}^2$ for each factor showing the percentage of explained variability in Carnitine in (A) FAMILY and (B) START cohort

Statistical significance was based on hierarchical linear models. * $p \leq 0.05$, **$p \leq 0.01$, ***$p \leq 0.001$

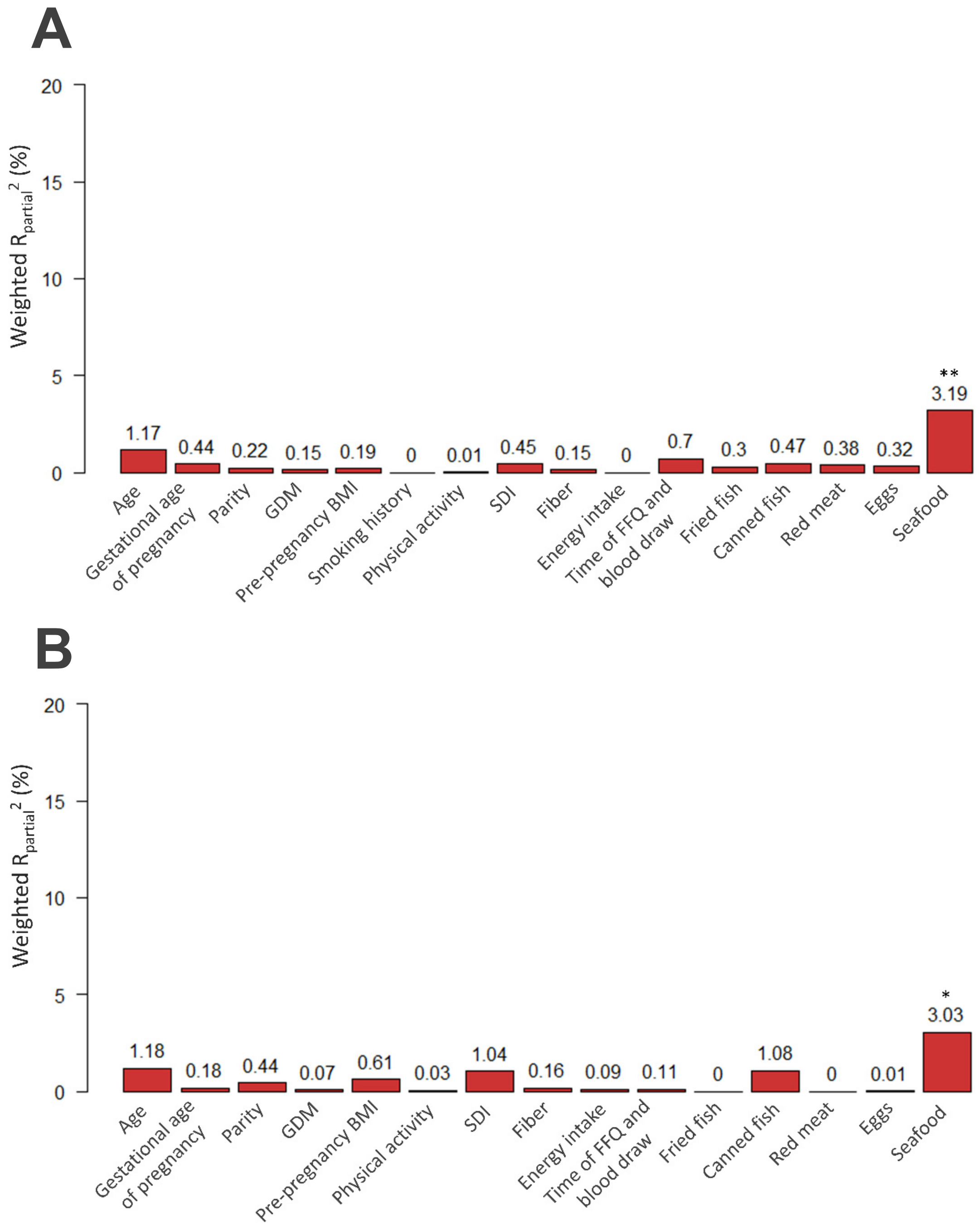

Figure S6: Weighted $R_{partial}^2$ for each factor showing the percentage of explained variability in trimethylamine *N*-oxide (TMAO) in (A) FAMILY and (B) START cohort

Statistical significance was based on hierarchical linear models. * $p \leq 0.05$, **$p \leq 0.01$

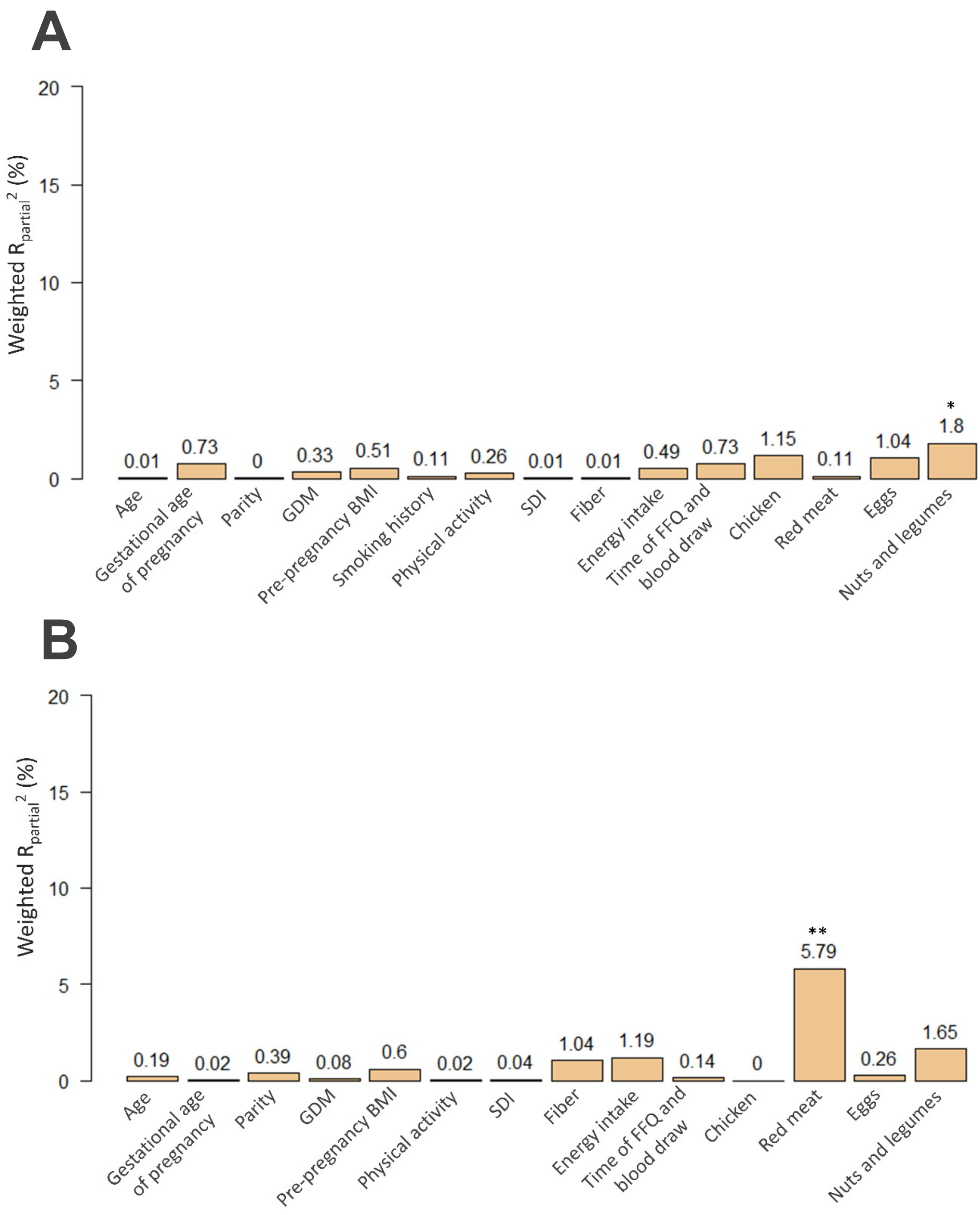

Figure S7: Weighted $R_{partial}^2$ for each factor showing the percentage of explained variability in 3-methylhistidine in (A) FAMILY and (B) START cohort

Statistical significance was based on hierarchical linear models. * $p \leq 0.05$, **$p \leq 0.01$

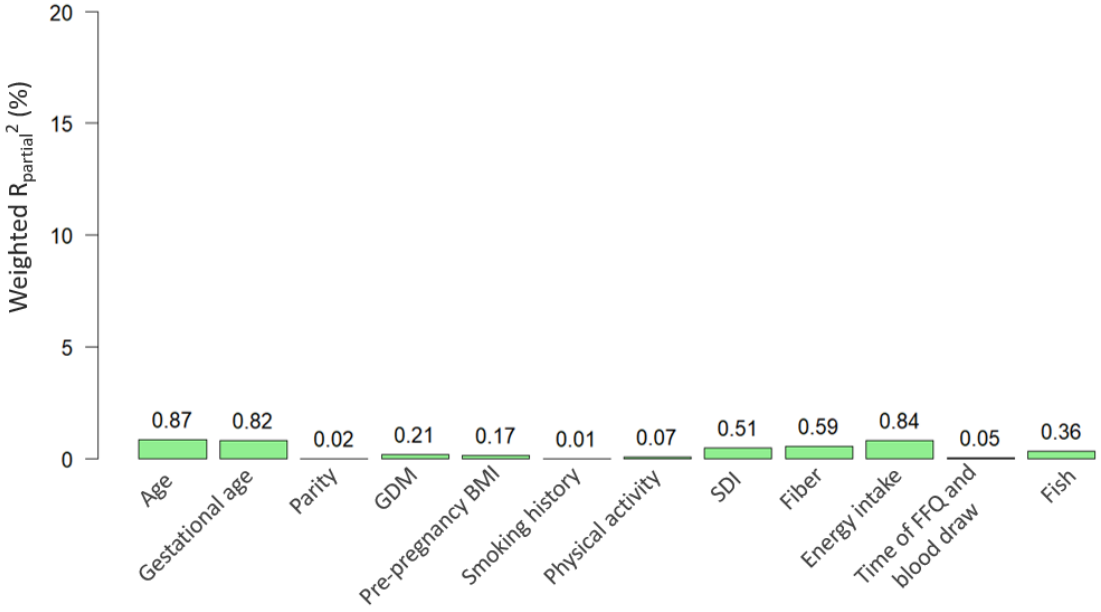

Figure S8: Weighted $R_{partial}^2$ for each factor showing the percentage of explained variability in Eicosapentaenoic acid (EPA, 20:5n-3) in FAMILY cohort

Statistical significance was based on ordinary least squares regression.

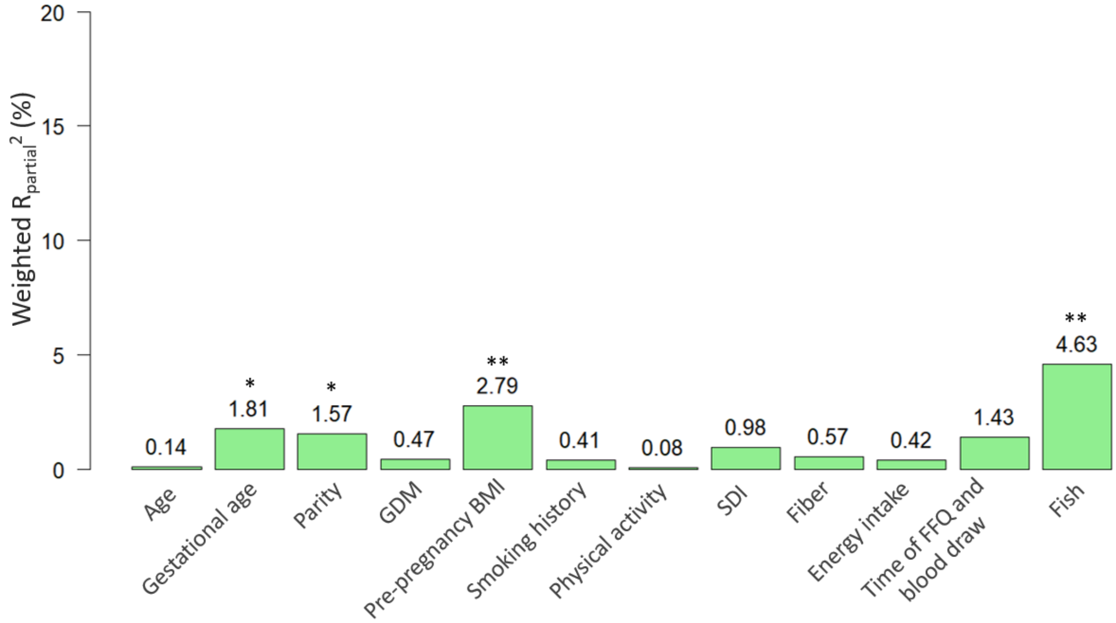

Figure S9: Weighted $R_{partial}^2$ for each factor showing the percentage of explained variability in Docosahexaenoic acid (DHA; 22:6n-3) in FAMILY cohort

Statistical significance was based on ordinary least squares regression. * $p \leq 0.05$, ** $p \leq 0.01$

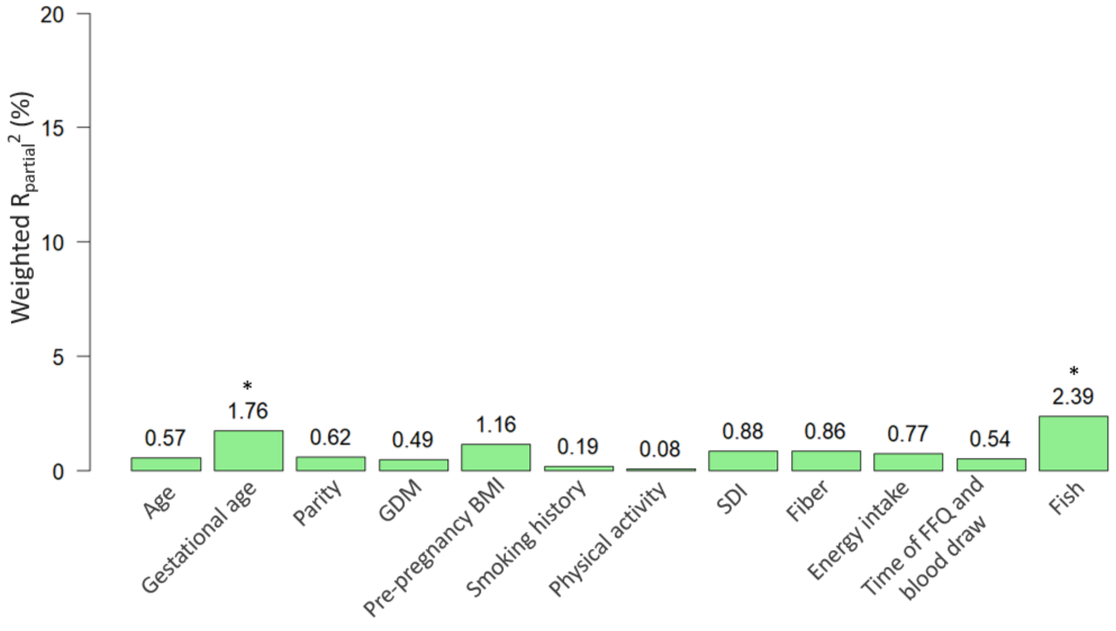

Figure S10: Weighted $R_{partial}^2$ for each factor showing the percentage of explained variability in EPA + DHA in FAMILY cohort

Statistical significance was based on ordinary least squares regression. * $p \leq 0.05$

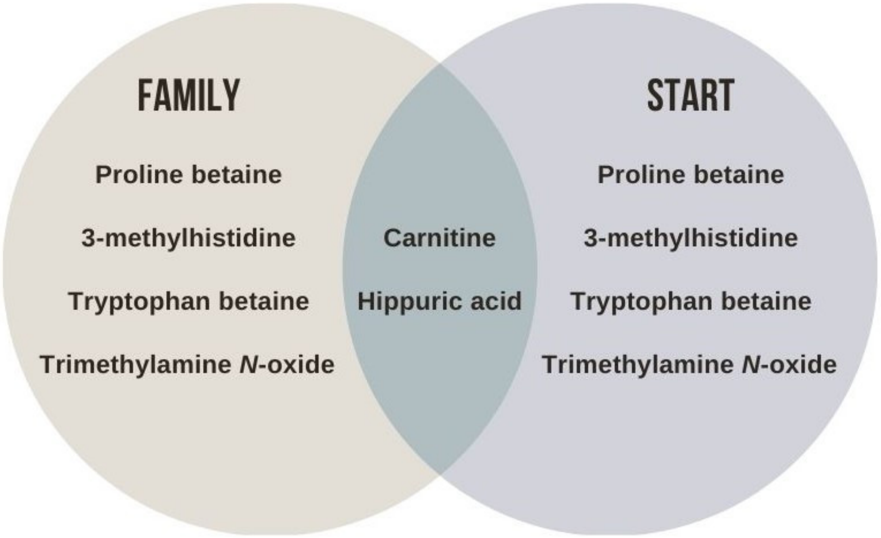

Figure S11: Venn diagram showing overlap of serum metabolites based on the cluster effect by ethnicity/cohort
